# The *Saccharomyces cerevisiae* AMPK, Snf1, Negatively Regulates the Hog1 MAPK Pathway in ER Stress Response

**DOI:** 10.1371/journal.pgen.1005491

**Published:** 2015-09-22

**Authors:** Tomoaki Mizuno, Yuto Masuda, Kenji Irie

**Affiliations:** Department of Molecular Cell Biology, Faculty of Medicine, University of Tsukuba, Tsukuba, Japan; Boston University Goldman School of Dental Medicine, UNITED STATES

## Abstract

Accumulation of unfolded proteins in the lumen of the endoplasmic reticulum (ER) causes ER stress. Snf1, the *Saccharomyces cerevisiae* ortholog of AMP–activated protein kinase (AMPK), plays a crucial role in the response to various environmental stresses. However, the role of Snf1 in ER stress response remains poorly understood. In this study, we characterize Snf1 as a negative regulator of Hog1 MAPK in ER stress response. The *snf1* mutant cells showed the ER stress resistant phenotype. In contrast, Snf1-hyperactivated cells were sensitive to ER stress. Activated Hog1 levels were increased by *snf1* mutation, although Snf1 hyperactivation interfered with Hog1 activation. Ssk1, a specific activator of MAPKKK functioning upstream of Hog1, was induced by ER stress, and its induction was inhibited in a manner dependent on Snf1 activity. Furthermore, we show that the *SSK1* promoter is important not only for Snf1-modulated regulation of Ssk1 expression, but also for Ssk1 function in conferring ER stress tolerance. Our data suggest that Snf1 downregulates ER stress response signal mediated by Hog1 through negatively regulating expression of its specific activator Ssk1 at the transcriptional level. We also find that *snf1* mutation upregulates the unfolded protein response (UPR) pathway, whereas Snf1 hyperactivation downregulates the UPR activity. Thus, Snf1 plays pleiotropic roles in ER stress response by negatively regulating the Hog1 MAPK pathway and the UPR pathway.

## Author Summary

All organisms are always exposed to several environmental stresses, including ultraviolet, heat, and chemical compounds. Therefore, every cell possesses defense mechanisms to maintain their survival under stressed conditions. Numerous studies have shown that a family of protein kinases plays a principal role in adaptive response to environmental stresses and perturbation of their regulation is implicated in a variety of human pathologies, such as cancer and neurodegenerative diseases. Elucidation of molecular mechanisms controlling their activities is still important not only for understanding how the organism acquires stress tolerance, but also for development of therapies for various diseases. In *Saccharomyces cerevisiae*, the Hog1 stress-responsive MAP kinase is activated by ER stress and coordinates a pleiotropic response to ER stress. However, the mechanisms for regulating Hog1 activity during ER stress response remain poorly understood. In this paper, we demonstrate that a *Saccharomyces cerevisiae* ortholog of mammalian AMP–activated protein kinase (AMPK), Snf1, negatively regulates Hog1 in ER stress response. ER stress induces expression of Ssk1, a specific activator of the Hog1 MAPK cascade. Snf1 lowers the expression level of Ssk1, thereby downregulating the signaling from upstream components to the Hog1 MAPK cascade. The activity of Snf1 is also enhanced by ER stress. Thus, our data suggest that Snf1 plays an important role in regulation of ER stress response signal mediated by Hog1.

## Introduction

The endoplasmic reticulum (ER) is the cellular organelle responsible for the folding and modification of newly synthesized secretory or membrane proteins. Environmental or developmental changes which perturb ER homeostasis, or genetic alterations causing production of irreversibly misfolded proteins lead to an accumulation of unfolded and misfolded proteins within the ER. This condition, which is collectively termed ER stress, is toxic to cells and has been implicated in a variety of human pathologies, such as diabetes, cancer and neurodegeneration, including Alzheimer, Parkinson and Huntington disease [1, 2]. Therefore, when ER stress is sensed, cells actuate adaptive signaling pathways to alleviate ER stress [1, 3]. In the budding yeast *Saccharomyces cerevisiae*, the unfolded protein response (UPR) signaling pathway, composed of an ER transmembrane protein Ire1 and a transcriptional activator Hac1, plays a principal role in ER stress response [1, 3]. When activated by ER stress, Ire1 excises the translation-inhibitory intron from *HAC1* mRNA, initiating splicing of *HAC1* mRNA and consequent production of Hac1 protein. Hac1 induces expression of target genes, such as genes encoding chaperones and proteins functioning ER-associated degradation, thus increasing the protein folding capacity of the ER. Although the UPR is undoubtedly essential for yeast cells to alleviate ER stress, a previous genome-wide study [4] has predicted that not less than 100 genes are involved in response to ER stress. Therefore, it remains to be fully elucidated how ER stress response is precisely controlled.

AMPK is evolutionarily conserved in eukaryotic cells and a key sensor of cellular energy status [5–7]. In *Saccharomyces cerevisiae*, a catalytic subunit of AMPK is encoded by the *SNF1* gene (S1 Fig). Similar to other members of the AMPK family, Snf1 forms a heterotrimeric complex with two regulatory subunits, the γ subunit Snf4 and one of the three alternative β subunits, Sip1, Sip2, or Gal83 [5]. The catalytic activity of Snf1 is regulated by phosphorylation at Thr-210 that is located in the activation loop of its kinase domain [8, 9]. Three upstream kinases, Sak1, Tos3, and Elm1, have been identified as kinases responsible for Snf1 activation [10–12]. Oppositely, Snf1 is inactivated by the Reg1-Glc7 protein phosphatase 1 complex; the catalytic subunit Glc7 is directed to Snf1 through the regulatory subunit Reg1 [13, 14]. Besides critical roles in adaptation to glucose deprivation and utilization of alternative carbon sources to glucose, the Snf1 complex is involved in the response to environmental stresses, such as heat and oxidative stresses [5, 15]. However, the role of Snf1 in ER stress response is as yet poorly understood.

The budding yeast Hog1, which is structurally highly similar to the mammalian p38 MAPK, was originally identified as a key protein kinase required for the adaptation of yeast cells to osmotic stress [16, 17]. In osmotic stress response, the Sln1-Ypd1-Ssk1 multistep phosphorelay system, which is homologous to bacterial two-component systems, regulates the Hog1 MAPK cascade (S2 Fig) [16, 17]. Under normal osmotic conditions, the membrane-associated histidine kinase Sln1 phosphorylates itself. The phosphate group is transferred to Ssk1 through the Ypd1 phosphotransmitter. Hyperosmotic stress inactivates Sln1, resulting in downregulation of the phosphorylation level of Ssk1. Dephosphorylated Ssk1 directly binds to and activates the Ssk2 and Ssk22 MAPKKKs, and consequently, leads to sequential activation of Pbs2 MAPKK and Hog1 MAPK. In addition to a pivotal role in osmotic stress response, Hog1 has been revealed as a regulator of a wide array of stress responses, including cold, heat and ER stresses [16–19]. In ER stress response, Hog1 is activated in an Ssk1-dependent manner [18]. However, the mechanisms that control Hog1 activity in ER stress response are still poorly understood.

In this study, we identified Snf1 as a negative regulator of Hog1 in ER stress response. Cells lacking Snf1 have elevated levels of active Hog1, whereas upregulation of Snf1 activity reduces Hog1 activation. ER stress induces expression of Ssk1, but this induction is counteracted by Snf1. These results indicate that Snf1 modulates Hog1 activation by controlling the expression level of its activator Ssk1. We also demonstrated that loss of Snf1 leads to upregulation of the UPR pathway, whereas the UPR activity is downregulated in Snf1-activated cells. Thus, Snf1 negatively regulates the Hog1 MAPK pathway and the UPR pathway in ER stress response.

## Results

### The Snf1 complex negatively regulates ER stress response

**Fig 1:**
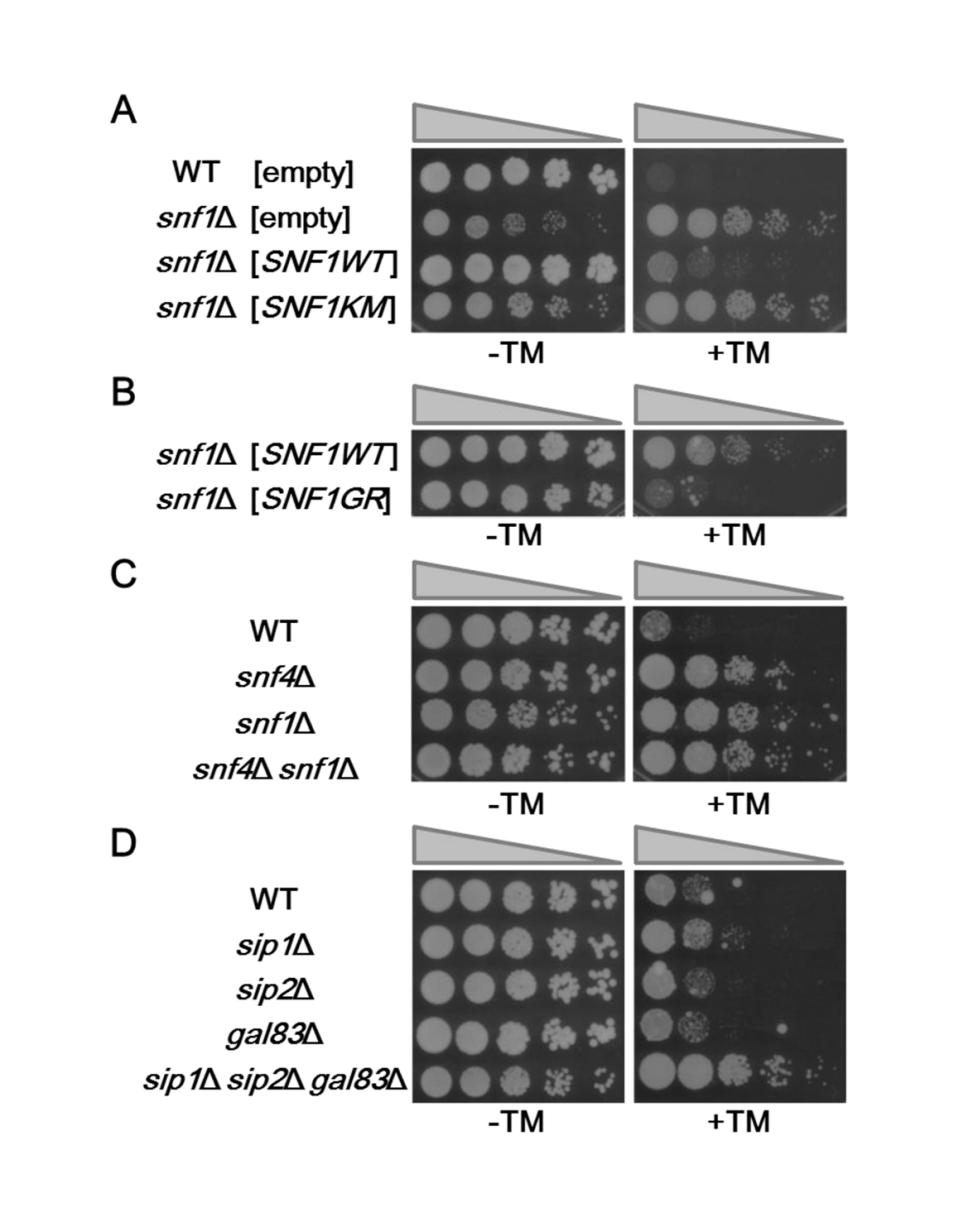
The yeast Snf1 pathway is involved in ER stress response. (A) ER stress resistance caused by deletion of the *snf1* gene. Wild-type (WT) and *snf1*Δ mutant strains harboring the indicated multicopy plasmids were spotted onto SD medium lacking or containing 1.5 μg/ml tunicamycin (TM) and incubated at 25°C. Wild-type Snf1 and Snf1(K84M) proteins were expressed in comparable amounts as shown in S3C Fig. (B) ER stress sensitivity caused by expression of the hyperactive form of Snf1. *snf1*Δ mutant strains harboring the indicated multicopy plasmids were spotted onto SD medium lacking or containing 1 μg/ml tunicamycin (TM) and incubated at 25°C. Wild-type Snf1 and Snf1(G53R) proteins were expressed in comparable amounts as shown in S3D Fig. (C) ER stress sensitivity caused by deletion of the *snf4* gene encoding the gamma subunit of the Snf1 complex. Wild-type (WT) and *snf4*Δ, *snf1*Δ, and *snf4*Δ *snf1*Δ mutant strains were spotted onto YPD medium lacking or containing 2 μg/ml tunicamycin (TM) and incubated at 25°C. (D) ER stress resistance caused by deletion of the genes encoding the beta subunits of the Snf1 complex. Wild-type (WT) and *sip1*Δ, *sip2*Δ, *gal83*Δ, and *sip1*Δ *sip2*Δ *gal83*Δ mutant strains were spotted onto YPD medium lacking or containing 1.5 μg/ml tunicamycin (TM) and incubated at 25°C.

In order to test whether the Snf1 protein kinase regulates ER stress response, cells carrying *snf1* deletion were plated on medium containing tunicamycin, a natural inhibitor of N-linked glycosylation that is widely employed as an inducer of ER stress. We unexpectedly found that compared to wild-type cells, the *snf1* mutant was resistant to tunicamycin (Fig 1A). To confirm that *snf1* mutation caused tunicamycin resistance, we transformed the *snf1* mutant with the plasmid that expresses *SNF1* and tested the transformants for growth on medium containing tunicamycin. Expression of *SNF1* significantly rescued the tunicamycin-resistant phenotype associated with the *snf1* mutation (Fig 1A and S3A Fig). To address the biological importance of Snf1 kinase activity, we generated a catalytically inactive form of Snf1 [Snf1(K84M)], in which Lys-84 in the ATP-binding motif was mutated to methionine [8]. When Snf1(K84M) was expressed in *snf1* mutants, the tunicamycin resistance was not rescued (Fig 1A). To examine the effect of Snf1 hyperactivation on ER stress response, we generated Snf1(G53R), a catalytically active form in which Gly-53 in the kinase domain has been mutated to arginine [8]. Expression of Snf1(G53R) resulted in hypersensitivity to tunicamycin (Fig 1B). These results indicate that Snf1 negatively regulates the response to ER stress in a manner dependent on its kinase activity.

We next asked if the regulatory subunits of Snf1 are involved in ER stress response. We found that deletion of the *SNF4* gene, which encodes the γ subunit of the Snf1 complex, caused increased resistance to tunicamycin (Fig 1C). Furthermore, cells harboring both *snf1* and *snf4* mutations displayed ER stress tolerance indistinguishable from that observed in the *snf1* single mutants (Fig 1C), indicating that Snf1 and Snf4 act in the same pathway. We next examined whether the β subunits, Sip1, Sip2, and Gal83, regulate ER stress response. We found that the *sip1 sip2 gal83* triple mutant was resistant to tunicamycin, although neither of their single mutants exhibited the obvious tunicamycin-resistant phenotype (Fig 1D). These results indicate that the Snf1 complex negatively regulates ER stress response.

### Phosphorylation of Snf1 is required for its role in ER stress response

**Fig 2:**
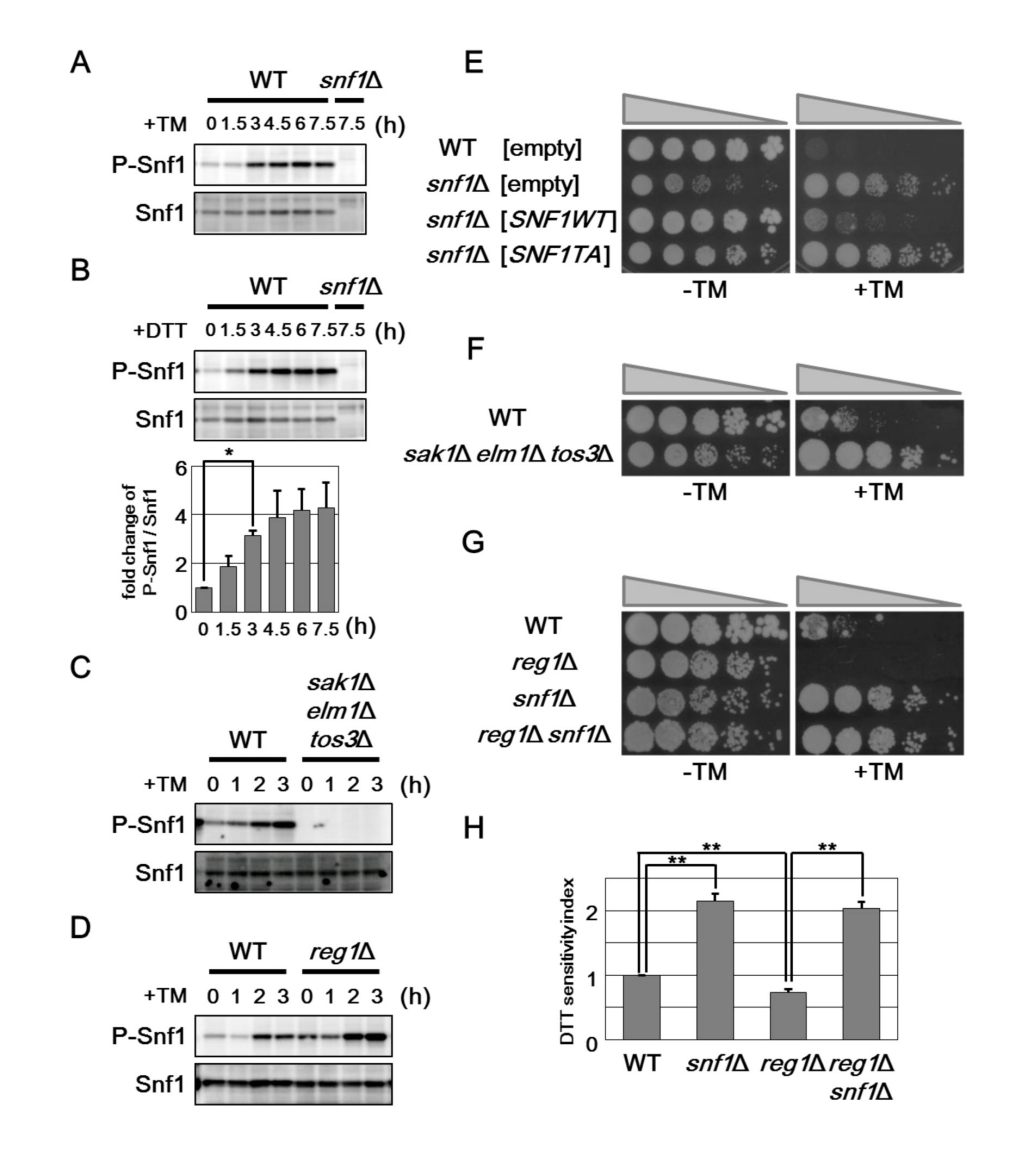
Snf1 phosphorylation is important for its role in ER stress response. (A) Effect of tunicamycin on Snf1 activity. Wild-type (WT) cells were grown at 25°C until exponential phase and treated with 2 μg/ml tunicamycin (TM) for the indicated time. Extracts prepared from each cell were immunoblotted with anti-phospho-AMPK (P-Snf1) and anti-Snf1 antibodies. (B) Effect of DTT on Snf1 activity. Wild-type (WT) cells were grown at 25°C until exponential phase and treated with 4 mM dithiothreitol (DTT) for the indicated time. Immunoblot was performed as described in (A). The intensities of phosphorylated Snf1 were measured and normalized to total Snf1 level. The values are plotted as the fold change from cells at the time of DTT addition. The data show mean ± SEM (n = 4). *P < 0.05 as determined by Student’s *t*-test. (C) Effects of the *sak1*Δ *tos3*Δ *elm1*Δ mutations on ER stress-induced Snf1 activation. Wild-type (WT) and *sak1*Δ *tos3*Δ *elm1*Δ mutant strains were grown at 25°C until exponential phase and treated with 2 μg/ml tunicamycin (TM) for the indicated time. Immunoblot was performed as described in (A). (D) Effects of the *reg1*Δ mutation on ER stress-induced Snf1 activation. Wild-type (WT) and *reg1*Δ mutant strains were analyzed as described in (C). (E) ER stress sensitivity in the *snf1*Δ mutants expressing a phospho-defective form of Snf1. Wild-type (WT) and *snf1*Δ mutant strains harboring the indicated multicopy plasmids were spotted onto SD medium lacking or containing 1.5 μg/ml tunicamycin (TM) and incubated at 25°C. Wild-type Snf1 and Snf1(T210A) proteins were expressed in comparable amounts as shown in S3E Fig. (F) ER stress resistance caused by deletion of the genes encoding the upstream kinases of the Snf1 complex. Wild-type (WT) and *sak1*Δ *tos3*Δ *elm1*Δ mutant strains were spotted onto YPD medium lacking or containing 1.5 μg/ml tunicamycin (TM) and incubated at 25°C. (G) ER stress sensitivity caused by deletion of the *reg1* gene. Wild-type (WT) and *reg1*Δ, *snf1*Δ, and *reg1*Δ *snf1*Δ mutant strains were spotted as described in (F). (H) DTT sensitivity of the *snf1*Δ and *reg1*Δ mutants. The mean of the DTT sensitivity index was shown with standard errors (n = 4). **P < 0.01 as determined by Student’s *t*-test.

Snf1 is phosphorylated at Thr-210 and activated by exposure of cells to alkaline pH and oxidative stresses [15]. Therefore, we investigated whether Snf1 is activated by treatment with ER stress. Anti-phospho AMPK antibodies that recognize the phosphorylated, activated form of AMPK were used to monitor phosphorylation of Snf1 at Thr-210 in the budding yeast [12]. In wild-type cells, we could detect Snf1 phosphorylation under unstressed conditions (Fig 2A). Treatment of cells with tunicamycin stimulated Snf1 phosphorylation within 1.5–3 hr, and its phosphorylation was persisted for at least 7.5 hr (Fig 2A). Similar observation was seen when cells were exposed to dithiothreitol (DTT), which causes ER stress by blocking disulfide bond formation in the ER (Fig 2B). Thus, ER stress induces activation of Snf1 through phosphorylation at the Thr-210 residue. We next asked whether ER stress-induced Snf1 activation is mediated by the upstream kinases, Sak1, Tos3, and Elm1. We found that *sak1 tos3 elm1* triple mutations completely abolished activation of Snf1 both in the presence or absence of ER stress (Fig 2C), although activated Snf1 levels were only slightly decreased in each single mutants (S4A and S4B Fig). This result indicates that ER stress induces Snf1 activation in a manner dependent on the three redundant kinases. Snf1 is inactivated through dephosphorylation mediated by the Reg1-Glc7 phosphatase complex. We next investigated the role of the Reg1-Glc7 complex in ER stress-induced Snf1 activation. Because the *glc7* deletion strain is lethal [20, 21], we used *reg1* deletion. Both in the presence or absence of tunicamycin treatment, phosphorylated Snf1 levels were clearly upregulated by *reg1* deletion (Fig 2D), indicating that the Reg1-Glc7 protein phosphatase 1 acts to inactivate Snf1 in ER stress response.

We next asked whether phosphorylation of Snf1 at Thr-210 is important for its function in ER stress response. We first examined the ability of Snf1(T210A), which contains a mutation of Thr-210 to Ala, to complement ER stress resistance associated with *snf1* deletion. The Snf1(T210A) mutant failed to complement the *snf1* defect (Fig 2E). We investigated whether the ER stress response involves the three upstream kinases and the phosphatase complex. We found that the *sak1 tos3 elm1* triple mutant cells, which were defective in Snf1 activation, were resistant to tunicamycin (Fig 2F). In contrast, *reg1* mutant cells in which Snf1 activity is upregulated exhibited hypersensitivity to tunicamycin; however, the stress sensitivity of the *reg1* mutant was completely suppressed by *snf1* deletion (Fig 2G). Similar results were obtained when DTT was used as an ER stressor (Fig 2H). Taken together, these results demonstrate that Snf1 is activated by ER stress through phosphorylation at Thr-210 and then negatively regulates ER stress response.

### Snf1 is involved in negative regulation of the UPR pathway

**Fig 3:**
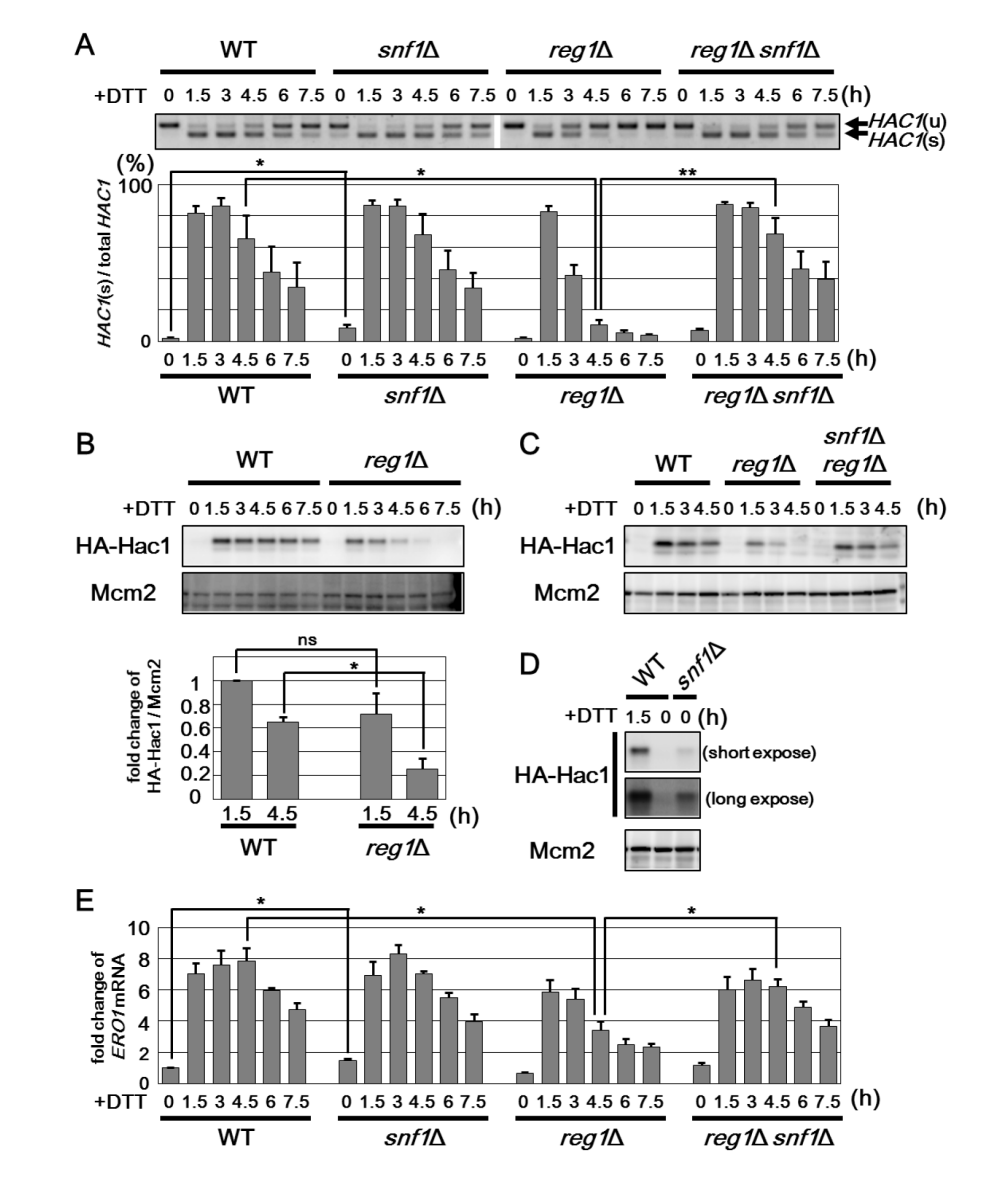
Snf1 negatively regulates the UPR pathway. (A) Splicing of *HAC1* mRNA in the *snf1*Δ and *reg1*Δ mutants. Wild-type (WT) and *snf1*Δ, *reg1*Δ and, *snf1*Δ *reg1*Δ mutant strains were grown at 25°C until exponential phase and treated with 4 mM dithiothreitol (DTT) for the indicated time. Total RNAs prepared from each strain were subjected to RT-PCR of *HAC1*. Positions of unspliced *HAC1* (*HAC1* ^*u*^) and spliced *HAC1* (*HAC1* ^*s*^) are indicated. The data show the mean of *HAC1* ^*s*^/(*HAC1* ^*u*^+ *HAC1* ^*s*^) with SEM (n = 4). *P < 0.05 and **P < 0.01 as determined by Student’s *t*-test. (B) Expression of Hac1 in the *reg1*Δ mutants. Wild-type (WT) and *reg1*Δ mutant strains harboring HA-tagged *HAC1* were grown at 25°C until exponential phase and treated with 4 mM dithiothreitol (DTT) for the indicated time. Extracts prepared from each strain were immunoblotted with anti-HA (HA-Hac1) and anti-Mcm2 antibodies. The intensities of HA-Hac1 were measured and normalized to the Mcm2 level. The values are plotted as the fold change from wild-type cells at 1.5 h after DTT addition. The data show mean ± SEM (n = 3). *P < 0.05 as determined by Student’s *t*-test. ns, not significant. (C) Expression of Hac1 in the *reg1*Δ *snf1*Δ mutants. Wild-type (WT) and *reg1*Δ and *reg1*Δ *snf1*Δ mutant strains harboring HA-tagged *HAC1* were grown at 25°C until exponential phase and treated with 4 mM dithiothreitol (DTT) for the indicated time. Immunoblot was performed as described in (B). (D) Expression of Hac1 in the *snf1*Δ mutants. Wild-type (WT) and *snf1*Δ mutant strains harboring HA-tagged *HAC1* were grown at 25°C until exponential phase and treated with 4 mM dithiothreitol (DTT) for the indicated time. Immunoblot was performed as described in (B). (E) Expression of *ERO1* gene in the *snf1*Δ and *reg1*Δ mutants. Wild-type (WT) and *snf1*Δ, *reg1*Δ, and *reg1*Δ *snf1*Δ mutant strains were grown at 25°C until exponential phase and treated with 4 mM dithiothreitol (DTT) for the indicated time. The mRNA levels were quantified by qRT-PCR analysis, and relative mRNA levels were calculated using *ACT1* mRNA. The data show mean ± SEM (n = 3). *P < 0.05 as determined by Student’s *t*-test.

Next, we explored the mechanism underlying the effect of Snf1 on ER stress response. Previous analyses in *Saccharomyces cerevisiae* have revealed that the UPR, composed of Ire1 and Hac1, is at the center of ER stress response [1, 3]. Therefore, we investigated a potential role for Snf1 in regulating the UPR. Upon ER stress, activated Ire1 excises the translation-inhibitory intron from *HAC1* mRNA, consequently producing Hac1 protein. Hac1 transcriptionally activates its target genes, including *KAR2* and *ERO1*. We first examined the kinetics of *HAC1* mRNA splicing (Fig 3A). In wild-type cells under unstressed conditions, the unspliced form of *HAC1* mRNA (*HAC1* ^*u*^) was robustly detected, but the spliced form (*HAC1* ^*s*^) was rarely detectable. Treatment of cells with ER stress promoted splicing of *HAC1* mRNA. The amount of *HAC1* ^*s*^ peaked 1.5 to 3 hr after DTT addition and gradually decreased thereafter. We next investigated the role of Snf1 in regulation of *HAC1* mRNA splicing using the *snf1* and *reg1* mutant cells. We found that both in *snf1* and *reg1* mutant cells, *HAC1* mRNA splicing was normally promoted in response to ER stress (Fig 3A). We found that downregulation of *HAC1* mRNA splicing was unaffected by *snf1* mutation. On the other hand, in the *reg1* mutant cells, *HAC1* ^*s*^ was decreased rapidly within 3 hr of DTT addition. Therefore, we compared the protein level of Hac1 between wild-type and the *reg1* mutant cells (Fig 3B). In wild-type cells, Hac1 protein was hardly detectable prior to ER stress treatment. Production of Hac1 was induced within 1.5 hr after exposure to DTT and subsequently downregulated. In the *reg1* mutant cells, Hac1 production was induced at levels comparable to that of wild-type cells. However, the amount of Hac1 declined rapidly within 3 to 4.5 hr after DTT treatment. A rapid decrease in Hac1 protein was also seen when cells harboring *reg1* mutation were exposed to tunicamycin (S5A Fig). Consistent with the protein level of Hac1, expression of the well-known Hac1 target genes, *ERO1* and *KAR2*, was reduced by *reg1* mutation (Fig 3E and S5B Fig). These UPR defects observed in the *reg1* mutant could be significantly restored by *snf1* mutation (Figs 3A, 3C and 3E and S5B). These results suggest that Snf1 participates in downregulation of the UPR pathway.

The observation that the UPR activity was downregulated by *reg1* mutation prompted us to perform a detailed analysis of the UPR activity in the *snf1* mutant cells. We found that the UPR activity under unstressed conditions was increased in the *snf1* mutant cells. In the absence of ER stress, the level of *HAC1* ^*s*^ in cells harboring *snf1* deletion was statistically higher than that in wild-type cells (Fig 3A). Consistent with this, the *snf1* mutant cells expressed a small amount of Hac1 protein even prior to treatment with ER stress (Fig 3D). Furthermore, under unstressed conditions, both *ERO1* and *KAR2* mRNAs were statistically significantly increased in the *snf1* mutant compared to wild-type cells (Fig 3E and S5B Fig). Taken together, these results indicate that Snf1 negatively regulates the UPR pathway.

### Snf1 acts as a negative regulator of Hog1 in ER stress response

**Fig 4:**
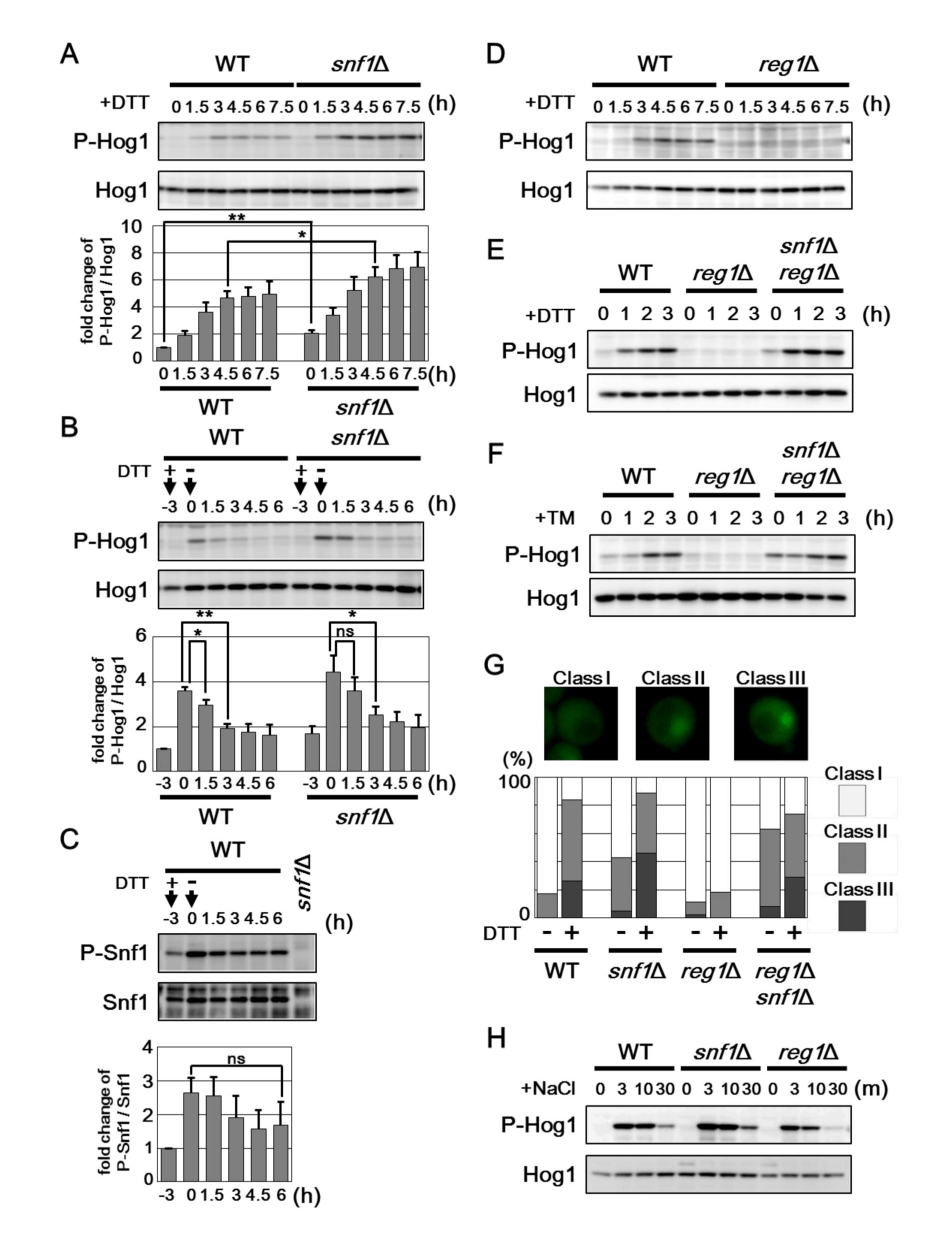
Snf1 inhibits Hog1 activation in ER stress response. (A) Effects of the *snf1*Δ mutation on ER stress-induced Hog1 activation. Wild-type (WT) and *snf1*Δ mutant strains were grown at 25°C until exponential phase and treated with 4 mM dithiothreitol (DTT) for the indicated time. Extracts prepared from each cell were immunoblotted with anti-phospho-p38 (P-Hog1) and anti-Hog1 antibodies. The intensities of phosphorylated Hog1 were measured and normalized to total Hog1 level. The values are plotted as the fold change from wild-type cells at the time of DTT addition. The data show mean ± SEM (n = 5). *P < 0.05 and **P < 0.01 as determined by Student’s *t*-test. (B) Hog1 activation is sustained in the *snf1*Δ mutant cells. Wild-type (WT) and *snf1*Δ mutant strains were grown at 25°C until exponential phase and treated with 4 mM dithiothreitol (DTT) for 3 hours. Then, each cell was recovered from ER stress by washing in medium lacking DTT and incubated further for the indicated time. Immunoblot was performed as described in (A). The intensities of phosphorylated Hog1 were measured and normalized to total Hog1 level. The values are plotted as the fold change from wild-type cells at the time of DTT addition. The data show mean ± SEM (n = 4). *P < 0.05 and **P < 0.01 as determined by Student’s *t*-test. ns, not significant. (C) Snf1 activation is sustained after removal of ER stress. Wild-type (WT) cells were grown at 25°C until exponential phase and treated with 4 mM dithiothreitol (DTT) for 3 hours. Then, each cell was recovered from ER stress by washing in medium lacking DTT and incubated further for the indicated time. Extracts prepared from each cell were immunoblotted with anti-phospho-AMPK (P-Snf1) and anti-Snf1 antibodies. The intensities of phosphorylated Snf1 were measured and normalized to total Snf1 level. The values are plotted as the fold change from cells at the time of DTT addition. The data show mean ± SEM (n = 4). The statistical difference was determined by Student’s *t*-test. ns, not significant. (D) Effects of the *reg1*Δ mutation on ER stress-induced Hog1 activation. Wild-type (WT) and *reg1*Δ mutant strains were analyzed as described in (A). (E, F) Effects of the *reg1*Δ and *snf1*Δ mutations on ER stress-induced Hog1 activation. Wild-type (WT) and *reg1*Δ and *reg1*Δ *snf1*Δ mutant strains were grown at 25°C until exponential phase and treated with 4 mM dithiothreitol (DTT) (E) or 2 μg/ml tunicamycin (TM) (F) for the indicated time. Immunoblot was performed as described in (A). (G) Effects of the *snf1*Δ and *reg1*Δ mutations on Hog1 localization. Wild-type (WT) and *snf1*Δ and *reg1*Δ mutant strains harboring GFP-tagged *HOG1* were treated with or without 4 mM dithiothreitol (DTT) for 2 h, and subjected to microscopy. The fluorescence intensities were measured, and then the ratios (N/C) of the fluorescence intensity per unit area in the nucleus/that in the cytoplasm were calculated. “Class I”, “Class II”, and “Class III” refer to cells in which a N/C ratio was < 1.25, 1.25–1.50, and > 1.50, respectively. Percentages of cells in each category are listed (n = 100). (H) Effects of the *snf1*Δ and *reg1*Δ mutations on sodium chloride stress-induced Hog1 activation. Wild-type (WT) and *snf1*Δ and *reg1*Δ mutant strains were grown at 25°C until exponential phase and treated with 0.4 M sodium chloride (NaCl) for the indicated time. Immunoblot was performed as described in (A).

The *snf1* mutation significantly enhanced resistance against ER stress, although UPR upregulation caused by *snf1* mutation was detected only under unstressed conditions. Therefore, additional mechanisms may contribute to ER stress resistance caused by *snf1* mutation. In the budding yeast, Hog1 MAPK is activated by ER stress through phosphorylation at critical threonine and tyrosine residues located in the activation loop [16, 17], and is in fact required for protecting cells against ER stress [18, 19]. Anti-phospho-p38 antibodies that recognize the phosphorylated form of mammalian p38 MAPK can be used to detect activated Hog1 in the budding yeast [22]. As observed previously [18], western blot analysis with anti-phospho-p38 antibody marginally detected the activated form of Hog1 in wild-type cells and its abundance was increased by treatment of cells with DTT (Fig 4A). To investigate the role of Snf1 in regulation of Hog1 activity, we monitored the activated form of Hog1 in the *snf1* mutant following induction of ER stress. We found that activated Hog1 levels were increased in *snf1* mutant cells both in the presence or absence of DTT treatment (Fig 4A). Similar results were obtained when cells were exposed to tunicamycin (S6A Fig). These results suggest that Snf1 has the inhibitory effect on Hog1 activation. The observation that Snf1 is activated by ER stress prompted us to test whether Snf1 acts to downregulate Hog1 activity during recovery from ER stress. We observed that Snf1 remained active even after removal of DTT from the medium (Fig 4C). In contrast, DTT removal allowed reduction of Hog1 activity (Fig 4B). However, Hog1 activation was prolonged in cells lacking Snf1 (Fig 4B). These results suggest that ER stress-activated Snf1 participates in the process that Hog1 activity returns to the basal level. Furthermore, it is suggested that additional mechanisms function in Hog1 inactivation during recovery from ER stress, since Hog1 dephosphorylation after DTT removal was delayed, but occurred in *snf1* mutant cells. Next, we examined the effect of Snf1 hyperactivation on ER stress-induced Hog1 activation. Strikingly, activation of Hog1 in response to DTT was diminished by *reg1* mutation which increases Snf1 activity (Fig 4D), and this defect could be entirely restored by *snf1* mutation (Fig 4E). Similar results were obtained when cells were exposed to tunicamycin (Fig 4F and S6B Fig). Our finding that *reg1* mutation interferes with Hog1 activation in response to ER stress strongly suggests that Snf1 acts as a negative regulator of Hog1 in ER stress response.

Previous report showed that ER stress-activated Hog1 accumulated in the nucleus [18]. To investigate the effect of Snf1 on nuclear accumulation of Hog1 in response to ER stress, we used the strain which expresses Hog1 carboxyl-terminally tagged with GFP (Fig 4G). As observed previously [18], Hog1 was uniformly distributed in the nucleus and cytosol under normal conditions and became enriched in the nucleus after ER stress treatment. Loss of Snf1 slightly but significantly increased nuclear localization of Hog1 even in the absence of ER stress. In contrast, nuclear accumulation of Hog1 in response to ER stress was obviously decreased in the *reg1* mutant cells; however, this defect was clearly suppressed by *snf1* deletion. These observations support a role of Snf1 in negative regulation of Hog1 in ER stress response.

It has been well-characterized that yeast cells activate Hog1 when exposed to hyperosmotic extracellular environments [16, 17]. We therefore examines whether Snf1 might be involved in the osmotic stress response mediated by Hog1. In wild-type cells, activated Hog1 is robustly detectable within 3 min of NaCl treatment and then rapidly decreases by 30 min (Fig 4H). Hog1 activation in response to hyperosmotic stress appeared to be enhanced and reduced by *snf1* and *reg1* mutations, respectively (Fig 4H). These alterations are probably attributed to a potential role of Snf1 in inhibiting Hog1 activation. Indeed, *snf1* mutation elevated the basal activity of Hog1 (Fig 4A), and *reg1* mutation partially suppressed the lethality of *SLN1*- and *YPD1*-deleted cells in which Hog1 is constitutively activated (see below). However, we could not find that *reg1* mutation resulted in hypersensitivity to osmotic stress (S6C Fig). Therefore, it remains obscure whether Snf1-mediated Hog1 regulation is physiologically important for osmotic stress response.

### Relationship between the UPR, Hog1 and Snf1

**Fig 5:**
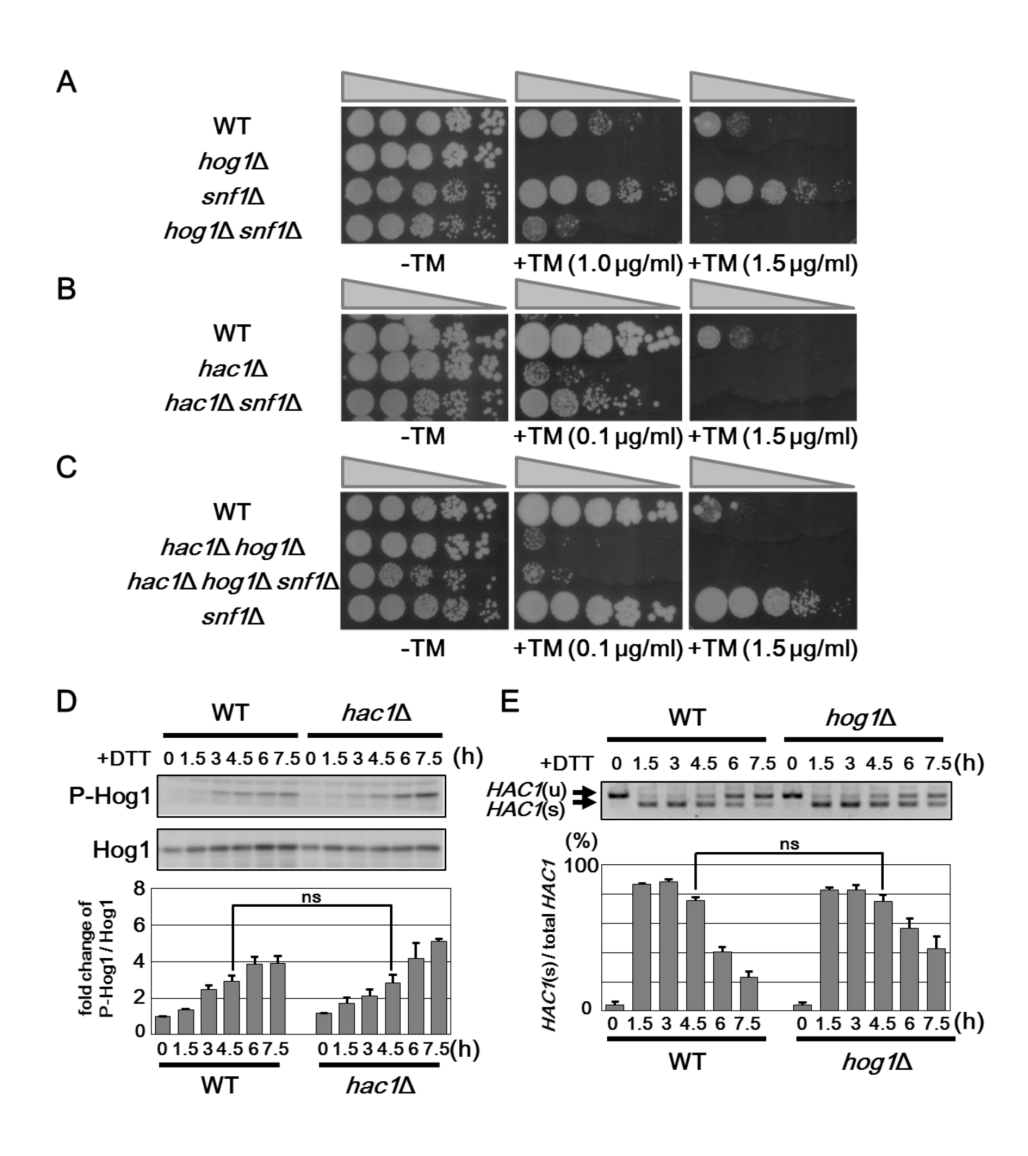
Snf1 regulates ER stress tolerance via Hog1 and UPR. (A) ER stress sensitivity in the *hog1*Δ *snf1*Δ mutants. Wild-type (WT) and *hog1*Δ, *snf1*Δ, and *hog1*Δ *snf1*Δ mutant strains were spotted onto YPD medium lacking or containing 1 or 1.5 μg/ml tunicamycin (TM) and incubated at 25°C. (B) ER stress sensitivity in the *hac1*Δ *snf1*Δ mutants. Wild-type (WT) and *hac1*Δ and *hac1*Δ *snf1*Δ mutant strains were spotted onto YPD medium lacking or containing 0.1 or 1.5 μg/ml tunicamycin (TM) and incubated at 25°C. (C) ER stress sensitivity in the *hac1*Δ *hog1*Δ *snf1*Δ mutants. Wild-type (WT) and *hac1*Δ *hog1*Δ, *snf1*Δ, and *hac1*Δ *hog1*Δ *snf1*Δ mutant strains were spotted onto YPD medium lacking or containing 0.1 or 1.5 μg/ml tunicamycin (TM) and incubated at 25°C. (D) Effects of the *hac1*Δ mutation on ER stress-induced Hog1 activation. Wild-type (WT) and *hac1*Δ mutant strains were grown at 25°C until exponential phase and treated with 4 mM dithiothreitol (DTT) for the indicated time. Extracts prepared from each cell were immunoblotted with anti-phospho-p38 (P-Hog1) and anti-Hog1 antibodies. The intensities of phosphorylated Hog1 were measured and normalized to total Hog1 level. The values are plotted as the fold change from wild-type cells at the time of DTT addition. The data show mean ± SEM (n = 3). The statistical difference was determined by Student’s *t*-test. ns, not significant. (E) Splicing of *HAC1* mRNA in the *hog1*Δ mutants. Wild-type (WT) and *hog1*Δ mutant strains were grown at 25°C until exponential phase and treated with 4 mM dithiothreitol (DTT) for the indicated time. Total RNAs prepared from each strain were subjected to RT-PCR of *HAC1*. Positions of unspliced *HAC1* (*HAC1* ^*u*^) and spliced *HAC1* (*HAC1* ^*s*^) are indicated. The data show the mean of *HAC1* ^*s*^/(*HAC1* ^*u*^+ *HAC1* ^*s*^) with SEM (n = 3). The statistical difference was determined by Student’s *t*-test. ns, not significant.

We next examined whether enhanced ER stress resistance in the *snf1* mutants is caused by Hog1 hyperactivation. We constructed the *snf1 hog1* double mutants and test them for growth on medium containing tunicamycin (Fig 5A). The *snf1 hog1* double mutant was sensitive to tunicamycin. However, we also found that ER stress sensitive phenotype of the *hog1* mutant could be partially suppressed by *snf1* mutation. As *snf1* mutation leads to upregulation of the UPR, we compared the effect of *snf1* deletion in cells having a wild-type, *hac1*, or *hac1 hog1* background. The *snf1* mutation modestly restored ER stress sensitivity caused by *hac1* mutation (Fig 5B). In contrast, the *snf1 hog1 hac1* triple mutants exhibited hypersensitivity to tunicamycin, similar to the *hog1 hac1* double mutants (Fig 5C), indicating that Hog1 and UPR are key targets of Snf1 in ER stress response.

As shown above, activities of the UPR and Hog1 pathways are upregulated by *snf1* deletion, but downregulated by *reg1* mutation which leads to Snf1 hyperactivation. These observations raised the possibility that Snf1 continuously regulates the UPR and Hog1 pathways. If this is true, we can observe the diminished Hog1 activation in *hac1* mutant cells or the reduced UPR activity in *hog1* mutant cells. First, we measured Hog1 activity in cells lacking Hac1. However, we could not find that loss of Hac1 reduced Hog1 activation (Fig 5D). We next monitored *HAC1* mRNA splicing in *hog1* mutant cells. In *hog1* mutant cells, *HAC1* mRNA splicing was normally induced, but retained longer than wild-type cells (Fig 5E). This is consistent with a previous observation [18] and indicates that *hog1* mutation does not reduce, but rather upregulates the UPR activity. Thus, the activities of the UPR and Hog1 pathways are independently regulated by Snf1.

### Snf1 negatively regulates Ssk1 expression

We next investigated how Snf1 negatively regulates Hog1 in ER stress response. The dephosphorylation of MAPK by protein phosphatases is well-known as a common mechanism for the negative regulation of the signaling mediated by MAPK [23]. Hog1 is dephosphorylated and inactivated by Ptp2 tyrosine phosphatase [24, 25]. Previously, it has been shown that loss of Ptp2 results in enhanced resistance to ER stress in a *HOG1*-dependent manner [19]. Therefore, we examined the relationship between Snf1 and Ptp2. In the *ptp2* mutant cells, basal activity of Hog1 was modestly increased and ER stress-induced Hog1 activation was significantly upregulated (S7A Fig). We found that Hog1 activation was enhanced in the *ptp2 snf1* double mutants compared with the *ptp2* mutant cells (S7A Fig), indicating that Snf1 negatively regulates Hog1 in ER stress response independently of Ptp2.

**Fig 6:**
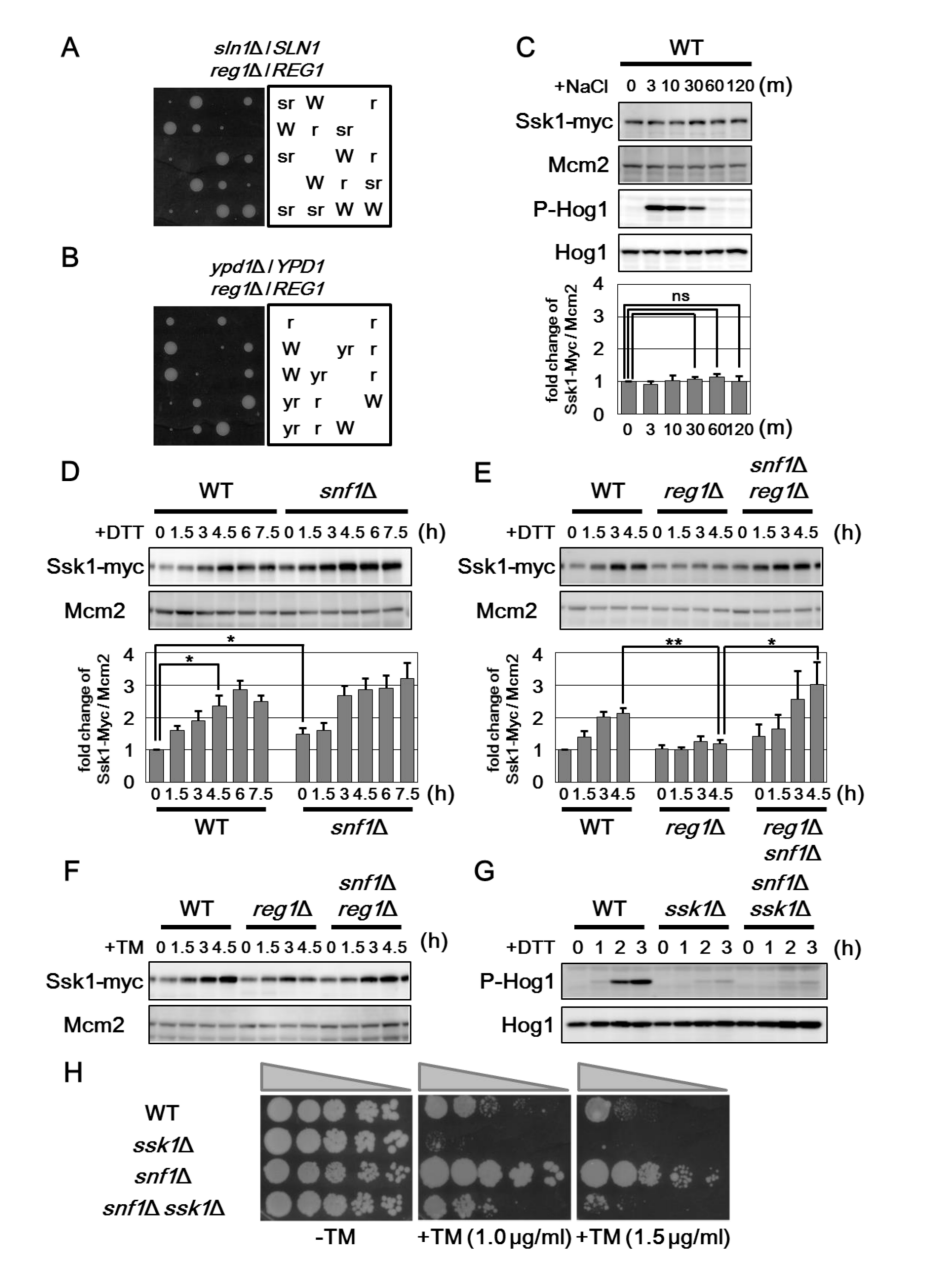
Snf1 downregulates the protein level of Ssk1. (A, B) Genetic interaction between the *ypd1*Δ, *sln1*Δ and *reg1*Δ mutations. Diploid *ypd1*Δ/*YPD1 reg1*Δ/*REG1* and *sln1*Δ/*SLN1 reg1*Δ/*REG1* yeast cells were sporulated, dissected on YPD plates and the meiotic products were incubated for 4 days at 25°C. Each genotype was shown in the right panel. Wild-type cells were labeled with W. The *ypd1*Δ, *sln1*Δ, and *reg1*Δ mutations were labeled with y, s, and r, respectively. (C) Effects of hyperosmotic stress on Ssk1 expression. Wild-type (WT) cells harboring Myc-tagged *SSK1* were grown at 25°C until exponential phase and treated with 0.4 M sodium chloride (NaCl) for the indicated time. Extracts prepared from each cell were immunoblotted with anti-Myc (Ssk1-Myc) and anti-Mcm2 antibodies. The intensities of Ssk1-Myc were measured and normalized to Mcm2 level. The values are plotted as the fold change from cells at the time of NaCl addition. The data show mean ± SEM (n = 4). The statistical difference was determined by Student’s *t*-test. ns, not significant. (D) Effects of the *snf1*Δ mutation on ER stress-induced upregulation of Ssk1. Wild-type (WT) and *snf1*Δ mutant strains harboring Myc-tagged *SSK1* were grown at 25°C until exponential phase and treated with 4 mM dithiothreitol (DTT) for the indicated time. Immunoblot was performed as described in (C). The intensities of Ssk1-Myc were measured and normalized to the Mcm2 level. The values are plotted as the fold change from wild-type cells at the time of DTT addition. The data show mean ± SEM (n = 4). *P < 0.05 as determined by Student’s *t*-test. (E) Effects of the *reg1*Δ and *snf1*Δ mutations on DTT-induced upregulation of Ssk1. Wild-type (WT) and *reg1*Δ and *reg1*Δ *snf1*Δ mutant strains harboring Myc-tagged *SSK1* were analyzed as described in (D). The data show mean ± SEM (n = 4). *P < 0.05 and **P < 0.01 as determined by Student’s *t*-test. (F) Effects of the *snf1*Δ and *reg1*Δ mutations on tunicamycin-induced upregulation of Ssk1. Wild-type (WT) and *reg1*Δ and *reg1*Δ *snf1*Δ mutant strains harboring Myc-tagged *SSK1* were grown at 25°C until exponential phase and treated with 2 μg/ml tunicamycin (TM) for the indicated time. Immunoblot was performed as described in (C). (G) Effects of the *ssk1*Δ and *snf1*Δ mutations on ER stress-induced Hog1 activation. Wild-type (WT) and *ssk1*Δ, and *snf1*Δ *ssk1*Δ mutant strains were grown at 25°C until exponential phase and treated with 4 mM dithiothreitol (DTT) for the indicated time. Extracts prepared from each cell were immunoblotted with anti-phospho-p38 (P-Hog1) and anti-Hog1 antibodies. (H) ER stress sensitivity in the *snf1*Δ *ssk1*Δ mutants. Wild-type (WT) and *ssk1*Δ, *snf1*Δ, and *snf1*Δ *ssk1*Δ mutant strains were spotted onto YPD medium lacking or containing 1 or 1.5 μg/ml tunicamycin (TM) and incubated at 25°C.

In ER stress response, signaling through the Hog1 pathway is controlled by the Sln1-Ypd1-Ssk1 phosphorelay system [18, 19]. Disruption of the *SLN1* gene results in lethality due to constitutive activation of Hog1 and, indeed, mutations in any of the four downstream genes, *SSK1*, *SSK2*, *PBS2*, and *HOG1*, suppress the *sln1* lethality by blocking activation of Hog1 [26, 27]. As shown above, Hog1 activity is considerably decreased in *reg1* mutant cells in which Snf1 is hyperactivated. Therefore, we tested whether deletion of the *REG1* gene suppresses the *sln1* lethality. We found that *reg1* mutation modestly suppressed the growth defect associated with *sln1* deletion (Fig 6A). Similarly, the lethality caused by *ypd1* deletion was partially suppressed by *reg1* mutation (Fig 6B). However, loss of Snf1 interfered with the ability of *reg1* mutation to suppress the *ypd1* lethality (S8A and S8B Fig). These results suggest that Snf1 regulates the component functioning downstream of Ypd1 in the Hog1 pathway.

In order to identify the molecule that mediates the signaling from Snf1 to Hog1, we examined the expression levels of components that act in the Hog1 pathway. We generated yeast strains carrying the carboxyl-terminally Myc-tagged genes, including *SSK1*, *SSK2*, *SSK22*, and *PBS2*, and analyzed their expression levels (Figs 6C–6E and S8C–S8E). Among them, we found that Ssk1 expression is changed by treatment with ER stress and genetic modulation of Snf1 signaling. In wild-type cells, the protein abundance of Ssk1 is increased following exposure to DTT and tunicamycin, but not NaCl (Fig 6C–6F), suggesting that ER stress specifically affects Ssk1 expression. The *snf1* mutation moderately increased Ssk1 expression (Fig 6D), suggesting that Ssk1 expression is negatively regulated by Snf1. Next, we examined the effect of Snf1 hyperactivation on the expression level of Ssk1. ER stress-mediated Ssk1 induction was effectively inhibited by *reg1* mutation that leads to hyperactivation of Snf1 (Fig 6E). This defect could be significantly restored by *snf1* mutation (Fig 6E). Similar results were obtained when cells were exposed to tunicamycin (Fig 6F). These results suggest that Ssk1 expression is negatively regulated by Snf1.

We next examined the functional importance of Ssk1 in Snf1-mediated regulation of Hog1 activity. As shown previously [18], activated Hog1 levels were significantly decreased in *ssk1* mutant cells (Fig 6G). This defect could not be suppressed by *snf1* mutation (Fig 6G), indicating that Ssk1 is important for Hog1 hyperactivation caused by *snf1* mutation. We also asked whether Ssk1 is involved in enhanced ER stress resistance of the *snf1* mutants. We found that *ssk1* mutation rendered cells lacking Snf1 sensitive to tunicamycin (Fig 6H). We also observed that the *ssk1 snf1* double mutant cells were more resistant to ER stress than the *ssk1* single mutants. ER stress tolerance of the *ssk1 snf1* double mutants is probably due to increased UPR activity caused by *snf1* mutation. Taken together, these results indicate that Snf1 inhibits Hog1 activation in response to ER stress by negatively regulating the expression level of Ssk1.

### Ssk1 expression is regulated by Snf1 at mRNA level

**Fig 7:**
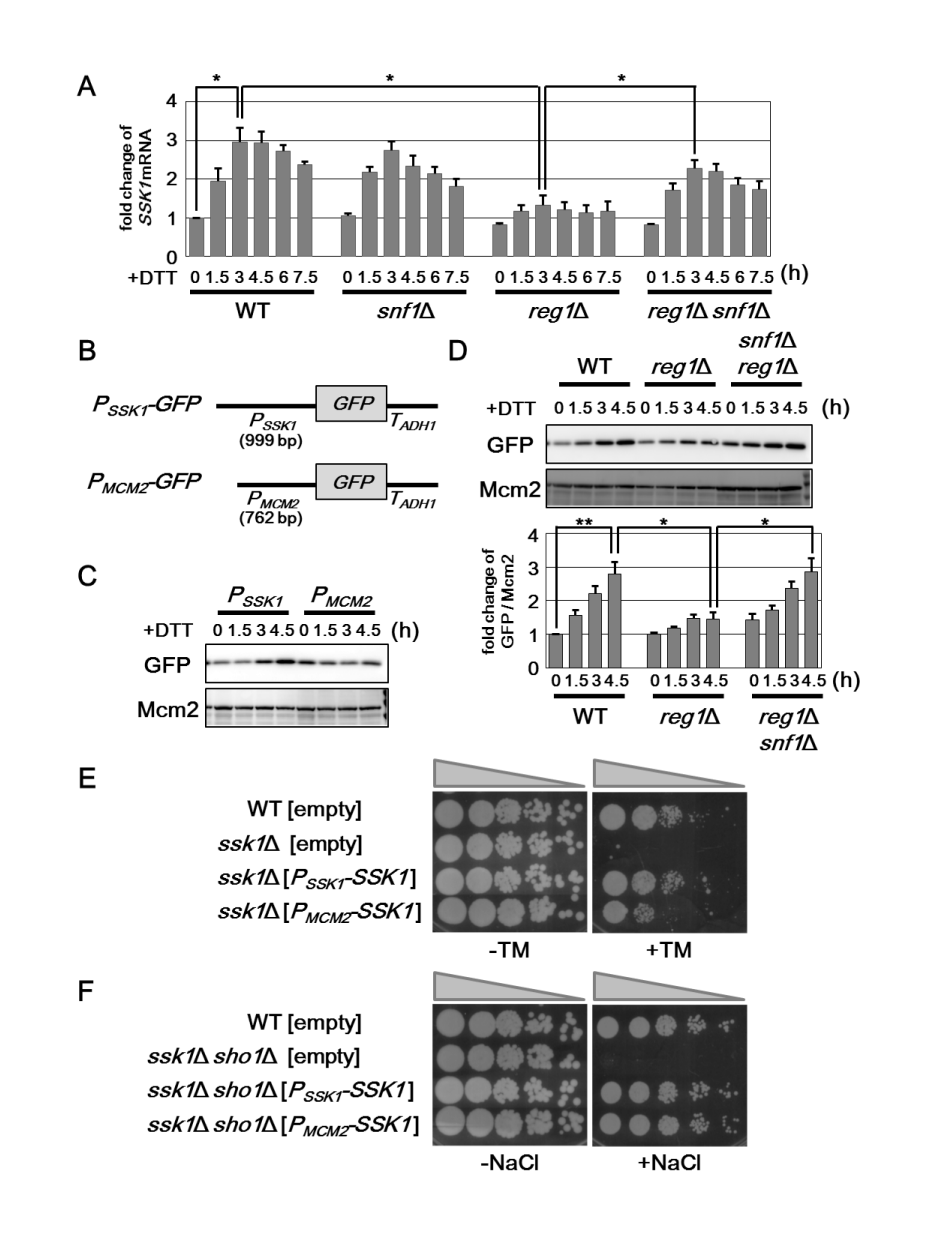
Snf1 negatively regulates the expression level of *SSK1* mRNA. (A) Effects of the *snf1*Δ and *reg1*Δ mutations on ER stress-induced upregulation of *SSK1* mRNA. Wild-type (WT) and *snf1*Δ, *reg1*Δ, and *reg1*Δ *snf1*Δ mutant strains were grown at 25°C until exponential phase and treated with 4 mM dithiothreitol (DTT) for the indicated time. The mRNA levels were quantified by qRT-PCR analysis, and relative mRNA levels were calculated using *ACT1* mRNA. The data show mean ± SEM (n = 3). *P < 0.05 as determined by Student’s *t*-test. (B) Schematic representation of the structure of *P*_*SSK1*_ *-GFP* and *P*_*MCM2*_ *-GFP*. (C) Effects of ER stress on expression of *P*_*SSK1*_ *-GFP* and *P*_*MCM2*_ *-GFP* reporters. Wild-type (WT) cells harboring the indicated plasmids were grown at 25°C until exponential phase and treated with 4 mM dithiothreitol (DTT) for the indicated time. Extracts prepared from each cell were immunoblotted with anti-GFP and anti-Mcm2 antibodies. (D) Effects of the *snf1*Δ and *reg1*Δ mutation on *SSK1* promoter activity. Wild-type (WT) and *reg1*Δ and *reg1*Δ *snf1*Δ mutant strains harboring the integration which expresses GFP under the control of *SSK1* promoter were grown at 25°C until exponential phase and treated with 4 mM dithiothreitol (DTT) for the indicated time. Extracts prepared from each cell were immunoblotted with anti-GFP and anti-Mcm2 antibodies. The intensities of GFP were measured and normalized to Mcm2 level. The values are plotted as the fold change from wild-type cells at the time of DTT addition. The data show mean ± SEM (n = 4). *P < 0.05 and **P < 0.01 as determined by Student’s *t*-test. (E) ER stress sensitivity in the *ssk1*Δ mutants. Wild-type (WT) and *ssk1*Δ mutant strains carrying the empty, *P*_*SSK1*_ *-SSK1*, or *P*_*MCM2*_ *-SSK1* plasmids were spotted onto YPD medium lacking or containing 1 μg/ml tunicamycin (TM) and incubated at 25°C. (F) Osmotic stress sensitivity in the *ssk1*Δ mutants. Wild-type (WT) and *ssk1*Δ *sho1*Δ mutant strains carrying the empty, *P*_*SSK1*_ *-SSK1*, or *P*_*MCM2*_ *-SSK1* plasmids were spotted onto YPD medium lacking or containing 1 M sodium chloride (NaCl) and incubated at 25°C.

To explore the mechanism by which Snf1 regulates the expression level of Ssk1, we measured the amount of *SSK1* mRNA by qRT-PCR (Fig 7A and S9A Fig). In wild-type cells, *SSK1* mRNA is increased following exposure to ER stress. This induction seemed to be normal in *snf1* mutant cells. On the other hand, *reg1* deletion significantly inhibited the induction of *SSK1* mRNA. This *reg1* defect could be restored by *snf1* mutation. These results indicate that Snf1 hyperactivation reduce the expression level of *SSK1* mRNA.

Numerous studies have revealed that Snf1 regulates the gene expression at the transcriptional level through phosphorylation of transcription factors [5, 6]. This raised the possibility that Snf1 regulates *SSK1* promoter activity. To test this possibility, we generated a *P*_*SSK1*_ *-GFP* reporter, consisting of the 5' upstream region of the *SSK1* gene to drive GFP expression (Fig 7B). Wild-type cells harboring the *P*_*SSK1*_ *-GFP* reporter displayed GFP expression in the absence of ER stress (Fig 7C). GFP expression from the *P*_*SSK1*_ *-GFP* reporter was increased following incubation with DTT (Fig 7C). On the other hand, we observed that DTT treatment had no obvious effect on expression of GFP derived from the *P*_*MCM2*_ *-GFP* reporter, in which the 5' upstream region of the *MCM2* gene is fused to *GFP* (Figs 7B and 7C and S9B). These results suggest that *SSK1* promoter is activated by ER stress. Next, we tested whether *P*_*SSK1*_ *-GFP* induction is regulated by the Snf1 pathway. In contrast to wild-type cells, *P*_*SSK1*_ *-GFP* expression was barely induced by DTT in *reg1* mutant cells (Fig 7D). This *reg1* defect could be significantly restored by *snf1* mutation (Fig 7D). These results strongly support the model in which Snf1 inhibits the activity of *SSK1* promoter.

We next examined whether *SSK1* induction in response to ER stress is important for resistance to ER stress. To test this, we generated two constructs, *P*_*SSK1*_ *-SSK1* and *P*_*MCM2*_ *-SSK1*, which express *SSK1* under the control of *SSK1* and *MCM2* promoters, respectively. Introduction of the *P*_*SSK1*_ *-SSK1* construct significantly rescued ER stress sensitive phenotype associated with *ssk1* mutation (Fig 7E). On the other hand, when the *P*_*MCM2*_ *-SSK1* construct was introduced into *ssk1* mutant cells, ER stress sensitivity was less effectively rescued (Fig 7E). This suggests that *SSK1* induction via its promoter activation is important for protecting cells against ER stress.

We also attempted to compare the ability of the *P*_*SSK1*_ *-SSK1* and *P*_*MCM2*_ *-SSK1* constructs to rescue the osmotic stress sensitivity caused by *ssk1* mutation. In osmotic stress response, the Hog1 pathway is activated by the membrane protein Sho1 in addition to Ssk1 [26]; hence, as shown in S9C Fig, the *ssk1 sho1* double mutants was sensitive to osmotic stress, although neither of their single mutants exhibited the obvious sensitivity to osmotic stress. Therefore, we transformed the *ssk1 sho1* double mutant cells with the *P*_*SSK1*_ *-SSK1* and *P*_*MCM2*_ *-SSK1* constructs and tested the transformants for growth under hyperosmotic conditions. We found that the *P*_*MCM2*_ *-SSK1* construct could rescue the osmotic stress sensitivity caused by *ssk1 sho1* mutations to same extent as the *P*_*SSK1*_ *-SSK1* construct (Fig 7F). Thus, it is unlikely that the *SSK1* promoter is involved in regulation of osmotic stress response. Taken together, the mechanisms underlying Hog1 activation mediated by Ssk1 are different between osmotic and ER stresses.

## Discussion

Previous studies have revealed that the *snf1* mutant cells exhibited hypersensitivity to a number of environmental stresses, including alkaline pH, heat shock, and genotoxic stress caused by hydroxyurea and methylmethane sulfonate [5]. Therefore, Snf1 was regarded as an essential regulator to confer resistance to environmental stresses. In this study, we unexpectedly found that the *snf1* mutants were resistant to ER stress. This finding not only indicates that Snf1 negatively regulates ER stress response, but also reveal a novel inhibitory role for Snf1 in stress response.

### The pleiotropic functions of Snf1 in ER stress response

Here, we revealed that Snf1 inhibits Hog1 activity by downregulation of the expression level of *SSK1* mRNA encoding an upstream activator of the Hog1 MAPK cascade. It is well-known that the dephosphorylation of MAPK by protein phosphatases is crucial for the negative regulation of the signaling mediated by MAPK [23]. The protein phosphatases, such as Ptc1, Ptp2, and Ptp3, play an important role in Hog1 inactivation [16, 17, 24, 25]. Previous report showed that Ptp2 and Ptp3 play the main and minor role, respectively, in negative regulation of Hog1 during ER stress response [19]. Nevertheless, why is Snf1 needed to function in downregulation of Hog1 activity? It is possible that Snf1 coordinates ER stress response with other cellular responses, since Snf1 is activated in the response to various environmental stresses [5, 6, 15]. Indeed, it has been reported that ER stress sensitivity is enhanced under the extracellular environments in which Snf1 activity is known to be elevated [28]. Alternatively, Snf1 may function to inactivate Hog1 in the whole cell level. Previous study showed that upon exposure to ER stress, Hog1 not only translocates into the nucleus and regulates the gene expression, but also functions in activation of autophagy in the cytoplasm [18]. On the other hand, Ptp2 and Ptp3 phosphatases are localized in the nucleus and cytoplasm, respectively [29]. Therefore, it is anticipated that Hog1 activity and its related cellular responses are negatively regulated in a manner different from the nucleus and cytoplasm. In contrast, Snf1 interferes with the signal from Ssk1 to Hog1 MAPK cascade through negative regulation of Ssk1 expression. Therefore, Snf1 is expected to contribute to downregulation of Hog1 in the whole cell level. Our analyses showed that loss of Snf1 moderately increased Hog1 activity, while Snf1 hyperactivation caused by *reg1* deletion effectively inhibited Hog1 activation. The existence of protein phosphatases for Hog1 may make apparently difficult to observe the effect of *snf1* mutation on Hog1 activity. It is well-known that expression of protein phosphatases for MAPK is induced by environmental stresses [23]. In fact, we found that Ptp2 was induced in response to ER stress (S7C Fig). Consistent with this, Hog1 inactivation after removal of ER stress was modestly delayed, but occurred in *snf1* mutant cells. Thus, it is likely that intricate signaling networks regulate Hog1 activity during ER stress response. Therefore, detailed analyses of the relationships between Hog1-mediated ER stress responses and the function of each negative regulator for Hog1 will be important for further understanding how Hog1 activity is controlled during ER stress response.

In this study, we observed that the *snf1* mutant cells expressed Hac1 even in the absence of ER stress. On the other hand, the expression level of Hac1 in the presence of ER stress was rapidly decreased in Snf1-hyperactivated cells. These observations suggest that Snf1 acts as a negative regulator of the UPR pathway. Although the activation mechanisms of the UPR pathway has been well-studied, there are only a few reports about how the UPR is inactivated after ER stress. Previously, two groups demonstrated the importance of the phosphorylation state of Ire1 kinase domain in the attenuation of the UPR activity [30, 31]. However, their proposed models are significantly different from each other. Therefore, the mechanisms by which the UPR is finally attenuated have yet to be elucidated. In the course of preparation of this manuscript, Casamayor and colleagues also reported that Snf1 is involved in yeast ER stress response [28]. Consistent with our findings, they showed that *reg1* mutation results in increased sensitivity to ER stress in a Snf1-dependent manner. Interestingly, they proposed the model in which Snf1 plays an inhibitory role in attenuation of the UPR by regulating the oligomerization of Ire1. In regard to the UPR activity in the *reg1* mutant cells, their results are distinctly different from our observations: they showed that increased UPR activity after ER stress treatment was prolonged in the *reg1* mutant cells; we found that in the *reg1* mutant cells, the UPR activity declined rapidly during ER stress response. The reason for this discrepancy is not clear now. However, this phenotypic distinction may be attributed to the difference in genetic background: their strains were derivatives of BY4741 and DBY746; we used W303 derivatives. Indeed, we could observe that the *snf1* mutant was resistant to tunicamycin, although they found no difference in ER stress sensitivity between wild-type and the *snf1* mutant cells. Thus, further analyses should be needed to elucidate the molecular mechanism by which Snf1 regulates the UPR signaling pathway.

We found here that *snf1* mutation increases the activities of the Hog1 and UPR pathways and leads to resistance to ER stress. Numerous studies have revealed that improper hyperactivation of stress-responsive signaling pathways is deleterious to cells and organisms [16, 23, 32]. In fact, constitutive activation of Hog1 under unstressed conditions causes cell lethality [26, 33]. We observed that cells deleted for both *SNF1* and *PTP2* genes showed a high basal activity of Hog1, but remains viable (S7B Fig). Thus, Hog1 activity in the *ptp2 snf1* double mutant cells is not high enough to induce lethality. We found that compared with wild-type cells, the *snf1* mutant cells possessed an increased Hog1 activity during ER stress response. However, it is noteworthy that a decline in the Hog1 activity occurred after removal of ER stress even in cells lacking Snf1. This indicates that the Hog1 activity in the *snf1* mutant cells is upregulated, but still remains under the control of the regulatory mechanisms. Therefore, it is possible that Hog1 upregulation caused by *snf1* mutation is preferable for yeast cells to survive in the presence of ER stress. Similar view may be applied to the UPR activity. Previous studies revealed that perturbation of the mechanism for properly attenuating Ire1 activity results in reduction of cell viability in the presence of ER stress [30, 31]. In the *snf1* mutant cells, the basal activity of UPR is significantly higher than wild-type cells; however, attenuation of the UPR was nearly unaffected by *snf1* mutation. Since the *snf1* mutant cells possesses high, but adjustable, UPR activity, loss of Snf1 may be preferable for cells to survive under ER stress. Thus, Snf1 plays pleiotropic roles in negative regulation of ER stress response.

### Snf1 negatively regulates Hog1 activation by downregulating Ssk1 expression

**Fig 8:**
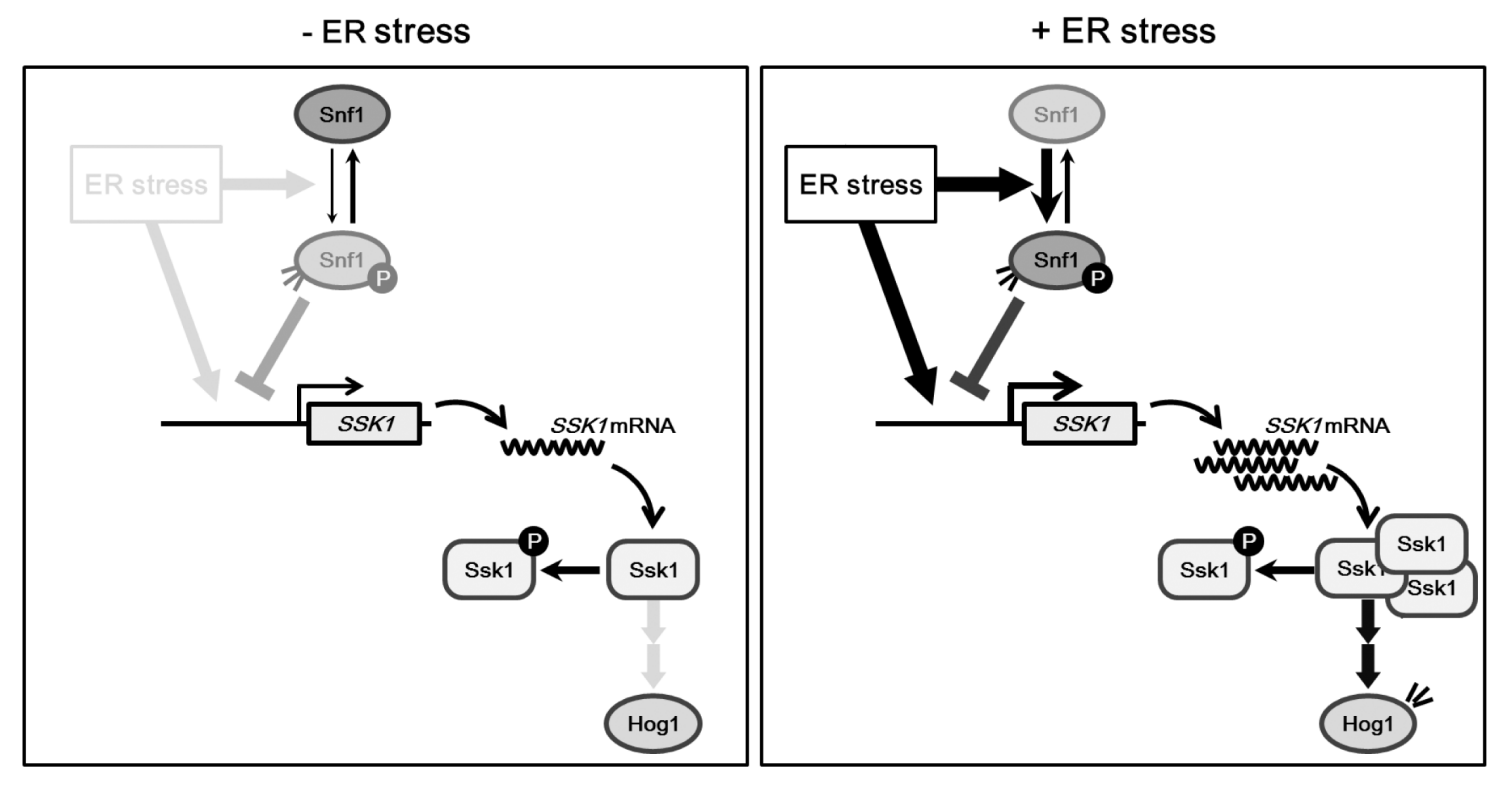
Proposed model for Snf1-mediated Hog1 regulation in ER stress response. Left panel. In the absence of ER stress, Ssk1 is inactivated through phosphorylation mediated by the upstream phosphorelay system. Right panel. In the presence of ER stress, increased expression of Ssk1 leads to accumulation of dephosphorylated Ssk1 and consequent activation of the Hog1 MAPK cascade. ER stress also induces the activation of Snf1. Activated Snf1 downregulates the signaling from Ssk1 by inhibiting Ssk1 expression.

The signaling through the Hog1 pathway is controlled by the Sln1-Ypd1-Ssk1 phosphorelay system [16, 17]. In osmotic stress response, Sln1 inactivation is a key step of Hog1 activation. Under normal osmotic conditions, active Sln1 leads to Ssk1 phosphorylation. Hyperosmotic stress inactivates Sln1, causing an increase of the dephosphorylated form of Ssk1. This promotes the Ssk1-Ssk2/Ssk22 physical interaction and results in activation of the Hog1 MAPK cascade. Previous studies demonstrated that Ssk1 is implicated in regulation of Hog1 activity during ER stress response [18, 19]. Little is understood, however, about the mechanism by which ER stress activates Hog1. In this study, we demonstrated that the expression level of Ssk1 is increased during ER stress response, and that elevation of Ssk1 protein level is important for cells to survive under ER stress conditions. In contrast, Ssk1 expression remained unchanged upon osmotic stress. These findings suggest that the mechanisms for the regulation of Hog1 are different among these different types of stress. Based on our data, we propose the model in which Hog1 activation in response to ER stress involves upregulation of Ssk1 (Fig 8). In unstressed conditions, Ssk1 is phosphorylated and inactivated by the upstream phosphorelay system. In the presence of ER stress, increased expression of Ssk1 overwhelms the phosphorylation activity of upstream phosphorelay system, leading to accumulation of dephosphorylated Ssk1 and consequent activation of the Hog1 MAPK cascade.

It is well-known that many protein kinases of the MAPKKK family can be activated by binding of their activators, similar to the budding yeast Ssk2 and Ssk22 MAPKKKs [23]. In unstimulated cells, MAPKKK is kept catalytically inactive through an autoinhibitory interaction between the regulatory domain and the kinase domain. Upon stimulation, binding of an activator protein leads to the dissociation of the autoinhibitory domain from the kinase domain and a consequent activation of MAPKKK. Mammalian MTK1/MEKK4 is a stress responsive MAPKKK that is structurally highly similar to the yeast Ssk2/Ssk22 and locates upstream in the p38 pathway [34, 35]. Previous studies have identified the GADD45 family proteins (GADD45α, GADD45β and GADD45γ) as MTK1/MEKK4 activators [36]. GADD45 binds to the autoinhibitory domain of MTK1/MEKK4 and relieve autoinhibition. Furthermore, each GADD45 exhibits distinct tissue expression patterns and is induced by a certain subset of environmental stresses, showing functional distinction among the GADD45 isoforms in p38 activation [36]. Our data presented here suggest that ER stress activates the Hog1 MAPK cascade by induction of Ssk1, in a manner similar to activation of p38 MAPK cascade though stress-mediated induction of GADD45.

To date, there is little understanding of the mechanism that controls the expression level of Ssk1. In this study, we show that Snf1 acts as a negative regulator of *SSK1* expression in ER stress response. We also demonstrate that the *SSK1* promoter is important for Snf1 to negatively regulate the mRNA level of *SSK1*. Snf1 phosphorylates a large number of transcription factors, and influences the transcription of hundreds of genes, including those involved in the utilization of alternate carbon sources and the metabolism of amino acids [5, 6]. Therefore, Snf1 is the most likely to inhibit the promoter activity of the *SSK1* gene through phosphorylation of the transcription factor. We also found that the expression level of Ssk1 protein was slightly different from that of *SSK1* mRNA. Ssk1 protein is more abundant in the *snf1* mutants than wild-type cells, although there was little difference in the expression level of *SSK1* mRNA between wild-type and the *snf1* mutant cells. This suggests that Snf1 inhibits Ssk1 expression at the translational or posttranslational levels. Intriguingly, it has been showed that the protein level of Ssk1 is negatively regulated by Ubc7 [37]. Ubc7 is an endoplasmic reticulum-associated ubiquitin-conjugating enzyme responsible for ER-associated degradation (ERAD). Snf1 may modulate Ssk1 degradation mediated by the ubiquitin-proteasome system involving Ubc7. Thus, identification of components downstream of Snf1, for example, which is responsible for induction of Ssk1 in response to ER stress, will provide valuable insights into the evolutionally conserved mechanism for regulation of the p38/Hog1 MAPK cascade.

## Materials and Methods

### Strains

Strains used in this study are listed in S1 Table. Yeast strains harboring the complete gene deletions and carboxyl-terminally Myc or GFP-tagged genes were generated by a PCR-based method as described previously [38]. All strains constructed by a PCR-based method were verified by PCR to confirm that replacement had occurred at the expected locus. Standard procedures were followed for yeast manipulations [39].

### Plasmids

Plasmids used in this study are described in S2 Table. In-Fusion cloning kits (Takara) was used to construct plasmids. The *P*_*SSK1*_ *-GFP* and *P*_*MCM2*_ *-GFP* plasmids were constructed as follows. The DNA fragment encoding GFP followed by the *ADH1* terminator (*T*_*ADH1*_) was obtained by PCR using the pFA6a-GFP vector [38] as a template. The *GFP-T*_*ADH1*_ DNA fragment was fused to 999-bp and 762-bp genomic fragments containing 5' upstream sequences of the *SSK1* and *MCM2* genes, yielding the *P*_*SSK1*_ *-GFP* and *P*_*MCM2*_ *-GFP* plasmids, respectively. Schemes detailing construction of plasmids and primer sequences are available on request.

### Protein extraction, western blot analysis and antibodies

Protein extracts were prepared essentially as described previously [40]. Briefly, cells grown to exponential phase were incubated with YPD or SD medium containing 2 μg/ml tunicamycin, 4 mM DTT or 0.4 M sodium chloride, for the indicated times. Cells were transferred into test tubes, mixed 1:1 with boiled medium, submerged in the boiling water for 3 min, and harvested by centrifugation. Cells were then subjected to a mild alkali treatment-based protein extraction method [41]. Western blot analysis was performed using the immunoreaction enhancer solution Can Get Signal (Toyobo) according to the manufacturer's protocol. Anti-HA monoclonal antibody 16B12 (Covance), anti-Myc monoclonal antibody 9E10 (Santa Cruz), anti-GFP monoclonal antibody JL-8 (Clontech), anti-phospho-p38 MAPK monoclonal antibody 28B10 (Cell Signaling), anti-phospho-AMPKα monoclonal antibody 40H9 (Cell Signaling), anti-Hog1 polyclonal antibody y-215 (Santa Cruz), anti-Snf1 polyclonal antibody yk-16 (Santa Cruz), and anti-Mcm2 polyclonal antibody N-19 (Santa Cruz) were used. Detection was carried out by using a LAS-4000 (Fuji Film) with Immobilon Westren (Merck Millipore). Signal intensities were quantified by ImageQuant (GE Healthcare), and statistical analysis was performed with Excel (Microsoft).

### RNA isolation and RT–PCR

Cells grown to exponential phase were incubated with YPD medium containing 2 μg/ml tunicamycin or 4 mM DTT, and harvested at the indicated times. Total RNA was then prepared using ISOGEN reagent (Nippon Gene) and the RNeasy Mini kit (Qiagen). First strands of cDNA were generated using the PrimeScript RT reagent Kit (Takara). The *HAC1* cDNA was amplified from first strands of cDNA with Blend Taq (TOYOBO), and then analyzed by agarose gel electrophoresis. Detection, quantification, and statistical analysis was carried out by using a LAS-4000 (Fuji Film), ImageQuant (GE Healthcare), and Excel (Microsoft), respectively. The cDNAs of *ERO1*, *KAR2*, and *SSK1*, were quantitated by a quantitative real-time RT-PCR (qRT-PCR) method using a 7500 fast real-time RT-PCR system (Applied Biosystems) with SYBR Premix Ex Taq (Takara). A standard curve was generated from diluted RNA derived from wild-type cells, and levels of gene expression were normalized to *ACT1* expression. *HAC1* primers (CTGGCTGACCACGAAGACGC and TTGTCTTCATGAAGTGATGA) were used to monitor splicing of *HAC1* mRNA. *ERO1* primers (TAACAGCAAATCCGGAACG and ACCAAATTTGACCAGCTTGC), *KAR2* primers (AGACTAAGCGCTGGCAAGCT and ACCACGAAAAGGGCGTACAG), *SSK1* primers (AGCTGGAAGCAGGGAGAAAG and TGAGTGAGGGTTGGAAGGTG), and *ACT1* primers (TGCCGAAAGAATGCAAAAGG and TCTGGAGGAGCAATGATCTTGA) were used to analyze the mRNA level of *ERO1*, *KAR2*, and *SSK1*.

### Stress sensitivity

Assays for tunicamycin toxicity were carried out as follows. Cells were grown to exponential phase, and cultures were adjusted to an optical density of 0.5. Cell cultures were then serially diluted 5-fold, spotted onto normal plates or plates containing the indicated concentrations of tunicamycin, followed by incubation at 25°C for 3 days (for plates lacking or containing 0.1 μg/ml tunicamycin), 5 days (for plates containing 0.5 μg/ml tunicamycin) and 7 days (for plates containing above 1 μg/ml tunicamycin). Assays for DTT toxicity were carried out as follows. Cells were grown to exponential phase, and cultures adjusted to an optical density of 0.05 were incubated with YPD medium or YPD medium containing 4 mM DTT for 12 h at 25°C. The sensitivity to DTT was estimated by dividing absorbance units in the presence of DTT by absorbance units in the absence of DTT, and then the ratios of DTT sensitivities of the mutants/wild-type were calculated as the DTT sensitivity index.

### Microscopy

To visualize GFP-tagged Hog1 in living cells, cells grown to exponential phase were incubated with SD medium containing 2 μg/ml tunicamycin or 4 mM DTT. Cells were then harvested at the indicated times, suspended in SD medium, and observed immediately using a Keyence BZ-X700 microscope (Keyence Corporation, Japan). Fluorescence intensities were quantified using Hybrid Cell Count BZ-H2C software (Keyence Corporation, Japan). To confirm nuclear localization of Hog1-GFP, cells were fixed for 10 min at 25°C by direct addition of 37% formaldehyde to a final concentration of 3.7%. Cells were then washed with PBS, stained with 4',6-diamidino-2-phenylindole (DAPI) and subjected to microscope observation. Images of Hog1-GFP in fixed cells were similar to those observed in living cells.

## Supporting Information

S1 Table Strains used in this study. (XLSX)

S2 Table Plasmids used in this study. (XLSX)

S1 Fig Schematic model of the Snf1 complex and its regulation. The encircled P’s represent phosphate groups. Proteins indicated with slashes represent functionally redundant components.
(TIF)

S2 Fig Schematic model of the Sln1-Ypd1-Ssk1 phosphorelay system and the Hog1 MAPK cascade. Arrows indicate positive signal flow, whereas blunt bars represent negative regulation. The double horizontal bars represents the plasma membrane. The encircled P’s represent phosphate groups. Proteins indicated with slashes represent functionally redundant components.
(TIF)

S3 Fig Snf1 negatively regulates ER stress response. (A) ER stress resistance caused by deletion of the *snf1* gene. Wild-type (WT) and *snf1*Δ mutant strains harboring the indicated centromeric plasmids were spotted onto SD medium lacking or containing 1.5 μg/ml tunicamycin (TM) and incubated at 25°C. Snf1 expression levels are shown in S3B Fig. (B-E) The expression levels of Snf1. Wild-type (WT) and *snf1*Δ mutant strains harboring the indicated plasmids were grown at 25°C until exponential phase. Extracts prepared from each cell were immunoblotted with anti-Snf1, anti-Mcm2 and anti-phospho-AMPK (P-Snf1) antibodies.
(TIF)

S4 Fig Effects of the *tos3*Δ, *sak1*Δ and *elm1*Δ mutations on Snf1 phosphorylation. (A) Effects of the *tos3*Δ and *sak1*Δ mutations on Snf1 phosphorylation. Wild-type (WT) and *tos3*Δ and *sak1*Δ mutant strains were grown at 25°C until exponential phase and treated with 2 μg/ml tunicamycin (TM) for the indicated time. Extracts prepared from each cell were immunoblotted with anti-phospho-AMPK (P-Snf1) and anti-Snf1 antibodies. (B) Effects of the *sak1*Δ and *elm1*Δ mutations on Snf1 phosphorylation. Wild-type (WT) and *sak1*Δ and *elm1*Δ mutant strains were analyzed as described in (A).
(TIF)

S5 Fig Snf1 negatively regulates expression of Hac1 and its target gene. (A) Expression of Hac1 in the *reg1*Δ mutants. Wild-type (WT) and *reg1*Δ mutant strains harboring the *HA-HAC1* integration were grown at 25°C until exponential phase and treated with 2 μg/ml tunicamycin (TM) for the indicated time. Extracts prepared from each strain were immunoblotted with anti-HA (HA-Hac1) and anti-Mcm2 antibodies. (B) Expression of the *KAR2* gene in the *snf1*Δ and *reg1*Δ mutants. Wild-type (WT) and *snf1*Δ, *reg1*Δ, and *reg1*Δ *snf1*Δ mutant strains were grown at 25°C until exponential phase and treated with 4 mM dithiothreitol (DTT) for the indicated time. The mRNA levels were quantified by qRT-PCR analysis, and relative mRNA levels were calculated using *ACT1* mRNA. The data show mean ± SEM (n = 3). *P < 0.05 and **P < 0.01 as determined by Student’s *t*-test.
(TIF)

S6 Fig Snf1 negatively regulates Hog1 activation. (A) Effects of the *snf1*Δ mutation on ER stress-induced Hog1 activation. Wild-type (WT) and *snf1*Δ mutant strains were grown at 25°C until exponential phase and treated with 2 μg/ml tunicamycin (TM) for the indicated time. Extracts prepared from each cell were immunoblotted with anti-phospho-p38 (P-Hog1) and anti-Hog1 antibodies. (B) Effects of the *reg1*Δ mutation on ER stress-induced Hog1 activation. Wild-type (WT) and *reg1*Δ mutant strains were analyzed as described in (A). (C) Osmotic stress sensitivity in the *reg1*Δ mutants. Wild-type (WT) and *reg1*Δ and *hog1*Δ mutant strains were spotted onto YPD medium lacking or containing 1 M sodium chloride (NaCl) and incubated at 25°C.
(TIF)

S7 Fig Snf1 acts independently of the Ptp2 phosphatase. (A) Effects of the *ptp2*Δ *snf1*Δ mutations on ER stress-induced Hog1 activation. Wild-type (WT) and *ptp2*Δ, and *ptp2*Δ *snf1*Δ mutant strains were grown at 25°C until exponential phase and treated with 2 μg/ml tunicamycin (TM) for the indicated time. Extracts prepared from each cell were immunoblotted with anti-phospho-p38 (P-Hog1) and anti-Hog1 antibodies. (B) Genetic interaction between the *snf1*Δ and *ptp2*Δ mutations. Diploid *snf1*Δ/*SNF1 ptp2*Δ/*PTP2* yeast cells were sporulated, dissected on YPD plates and the meiotic products were incubated at 25°C. Each genotype was shown in the right panel. Wild-type and *snf1*Δ and *ptp2*Δ mutant cells were labeled with W, s, and p, respectively. (C) The expression level of Ptp2 during ER stress response. Wild-type (WT) cells harboring harboring Myc-tagged *PTP2* were grown at 25°C until exponential phase and treated with 2 μg/ml tunicamycin (TM) or 4 mM dithiothreitol (DTT) for the indicated time. Extracts prepared from each cell were immunoblotted with anti-Myc and anti-Mcm2 antibodies.
(TIF)

S8 Fig Relationship between Snf1 and the Hog1 pathway. (A, B) Genetic interaction between the *ypd1*Δ, *reg1*Δ and *snf1*Δ mutations. Diploid *ypd1*Δ/*YPD1* (A) and *ypd1*Δ/*YPD1 reg1*Δ/*REG1 snf1*Δ/*SNF1* (B) yeast cells were sporulated, dissected on YPD plates and the meiotic products were incubated at 25°C. Each genotype was shown in the right panel. Wild-type and *snf1*Δ, *reg1*Δ, and *ypd1*Δ mutant cells were labeled with W, s, r, and y, respectively. (C-E) The expression levels of Ssk2, Ssk22 and Pbs2 during ER stress response. Wild-type (WT) and *reg1*Δ, and *reg1*Δ *snf1*Δmutant strains harboring Myc-tagged *SSK2* (C), *SSK22* (D), or *PBS2* (E) were grown at 25°C until exponential phase and treated with 2 μg/ml tunicamycin (TM) for the indicated time. Extracts prepared from each cell were immunoblotted with anti-Myc and anti-Mcm2 antibodies.
(TIF)

S9 Fig Snf1 negatively regulates *SSK1* mRNA expression. (A) Effects of the *snf1*Δ and *reg1*Δ mutations on ER stress-induced upregulation of *SSK1* mRNA. Wild-type (WT) and *reg1*Δ and *reg1*Δ *snf1*Δ mutant strains were grown at 25°C until exponential phase and treated with 2 μg/ml tunicamycin (TM) for the indicated time. The mRNA levels were quantified by qRT-PCR analysis, and relative mRNA levels were calculated using *ACT1* mRNA. The data show mean ± SEM (n = 4). **P < 0.01 as determined by Student’s *t*-test. (B) Effects of ER stress on the *MCM2* promoter activity. Wild-type strain harboring a *P*_*MCM2*_ *-GFP* reporter plasmid were analyzed as described in Fig 6C. The intensities of GFP were measured and normalized to Mcm2 level. The values are plotted as the fold change from wild-type cells at the time of DTT addition. The data show mean ± SEM (n = 3). The statistical difference was determined by Student’s *t*-test. ns, not significant. (C) Osmotic stress sensitivity in the *sho1*Δ and *ssk1*Δ mutants. Wild-type (WT) and *sho1*Δ, *ssk1*Δ, and *ssk1*Δ *sho1*Δ mutant strains were spotted onto YPD medium lacking or containing 1 M sodium chloride (NaCl) and incubated at 25°C.
(TIF)
