# Supplementary figures and images for: The Saccharomyces cerevisiae AMPK, Snf1, Negatively Regulates the Hog1 MAPK Pathway in ER Stress Response
Source: PLoS Genet. 2015 Sep 22;11(9):e1005491. doi: 10.1371/journal.pgen.1005491 (PMC4578879; doi:10.1371/journal.pgen.1005491)

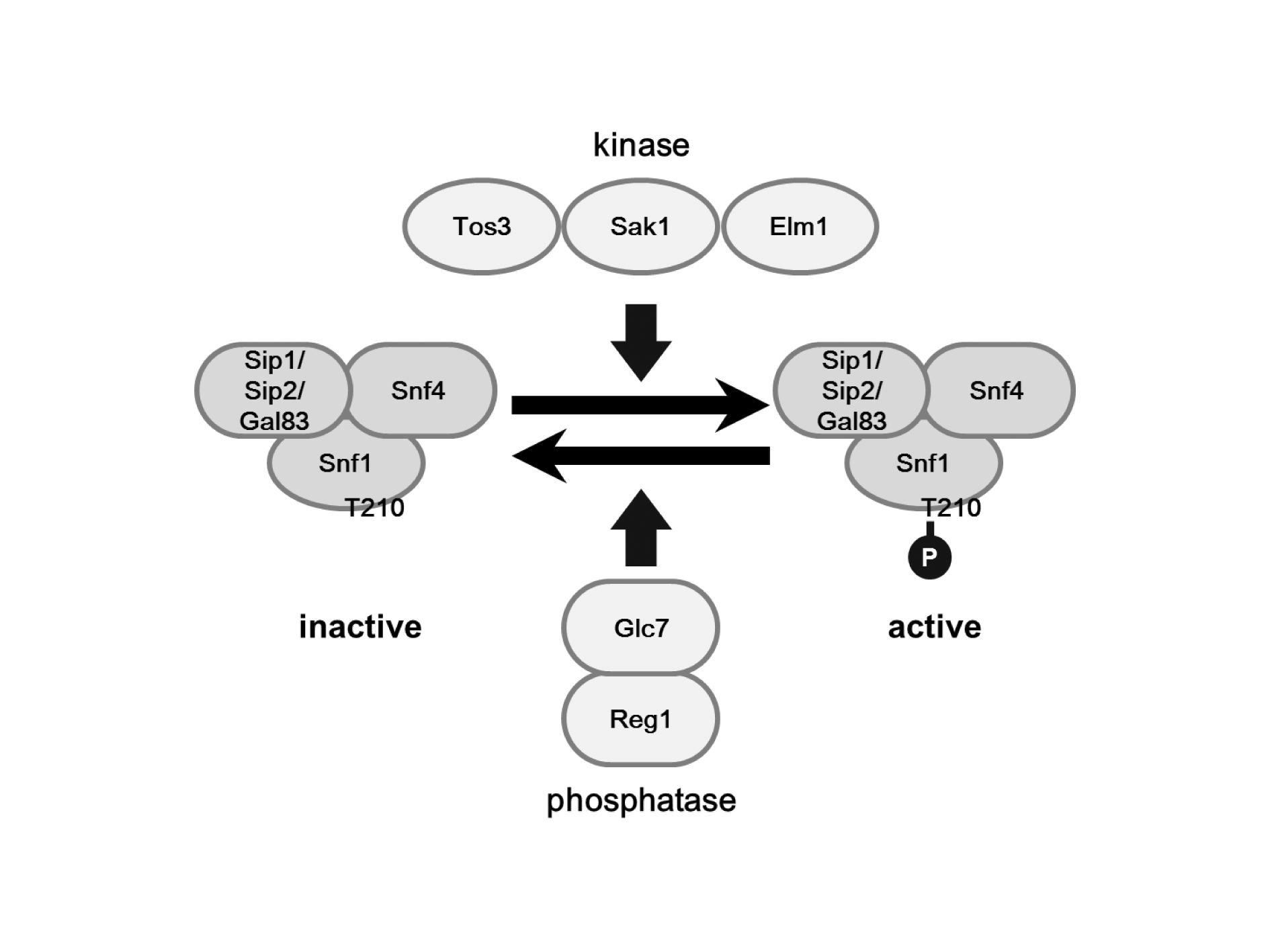

Supplement: S1 Fig — The encircled P’s represent phosphate groups. Proteins indicated with slashes represent functionally redundant components. (TIF) [file pgen.1005491.s003.tif]

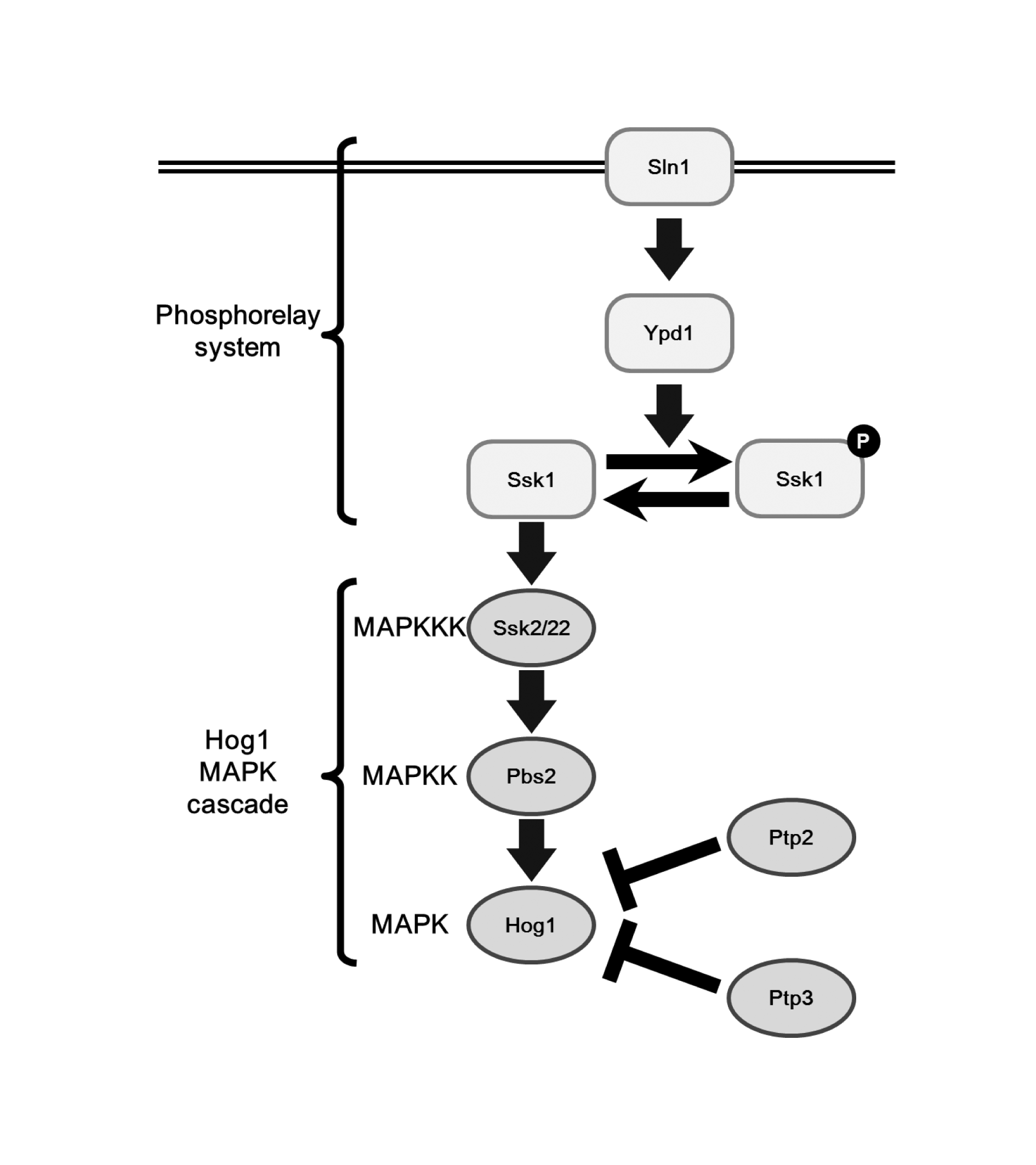

Supplement: S2 Fig — Arrows indicate positive signal flow, whereas blunt bars represent negative regulation. The double horizontal bars represents the plasma membrane. The encircled P’s represent phosphate groups. Proteins indicated with slashes represent functionally redundant components. (TIF) [file pgen.1005491.s004.tif]

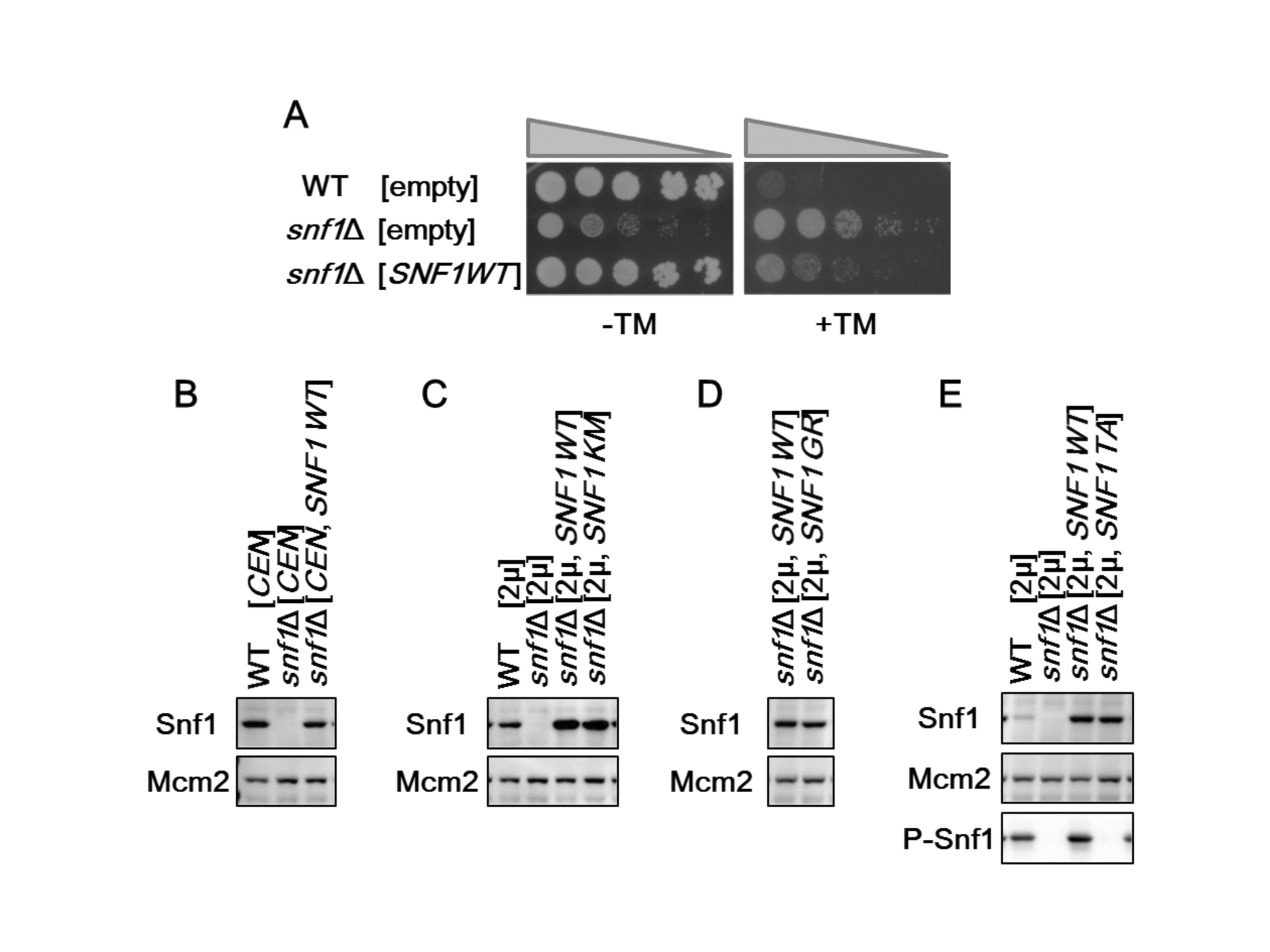

Supplement: S3 Fig — (A) ER stress resistance caused by deletion of the snf1 gene. Wild-type (WT) and snf1Δ mutant strains harboring the indicated centromeric plasmids were spotted onto SD medium lacking or containing 1.5 μg/ml tunicamycin (TM) and incubated at 25°C. Snf1 expression levels are shown in S3B Fig. (B-E) The expression levels of Snf1. Wild-type (WT) and snf1Δ mutant strains harboring the indicated plasmids were grown at 25°C until exponential phase. Extracts prepared from each cell were immunoblotted with anti-Snf1, anti-Mcm2 and anti-phospho-AMPK (P-Snf1) antibodies. (TIF) [file pgen.1005491.s005.tif]

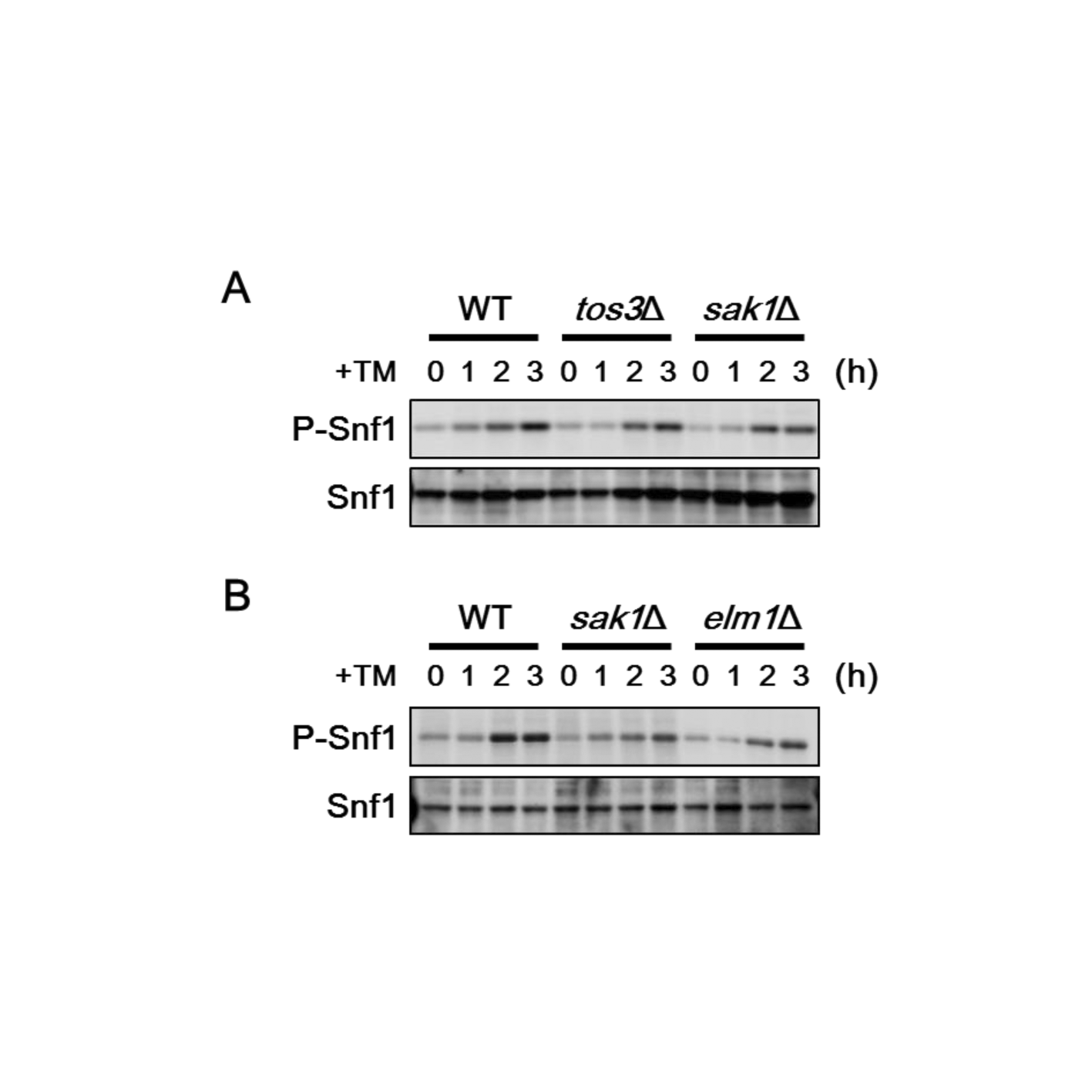

Supplement: S4 Fig — (A) Effects of the tos3Δ and sak1Δ mutations on Snf1 phosphorylation. Wild-type (WT) and tos3Δ and sak1Δ mutant strains were grown at 25°C until exponential phase and treated with 2 μg/ml tunicamycin (TM) for the indicated time. Extracts prepared from each cell were immunoblotted with anti-phospho-AMPK (P-Snf1) and anti-Snf1 antibodies. (B) Effects of the sak1Δ and elm1Δ mutations on Snf1 phosphorylation. Wild-type (WT) and sak1Δ and elm1Δ mutant strains were analyzed as described in (A). (TIF) [file pgen.1005491.s006.tif]

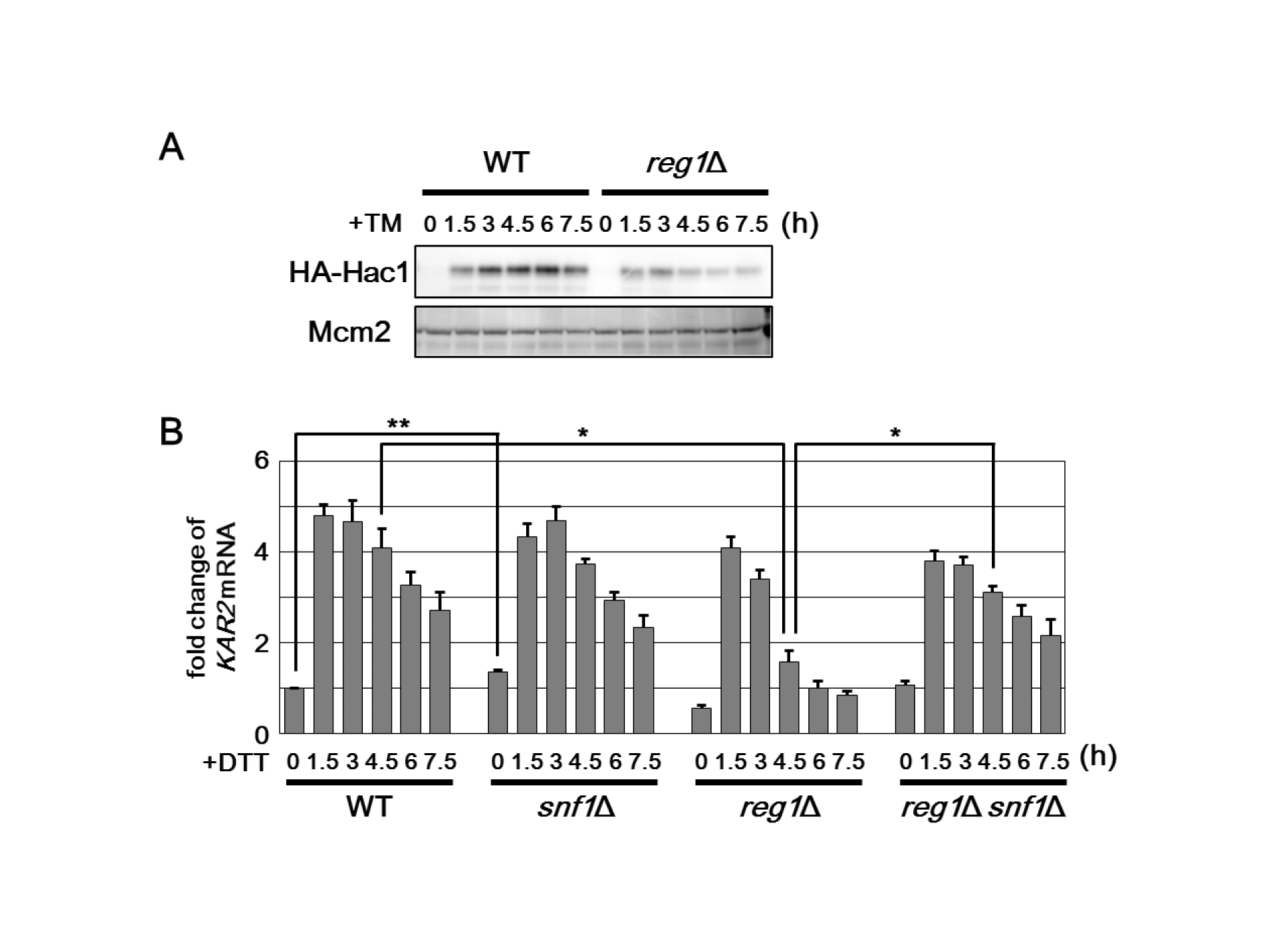

Supplement: S5 Fig — (A) Expression of Hac1 in the reg1Δ mutants. Wild-type (WT) and reg1Δ mutant strains harboring the HA-HAC1 integration were grown at 25°C until exponential phase and treated with 2 μg/ml tunicamycin (TM) for the indicated time. Extracts prepared from each strain were immunoblotted with anti-HA (HA-Hac1) and anti-Mcm2 antibodies. (B) Expression of the KAR2 gene in the snf1Δ and reg1Δ mutants. Wild-type (WT) and snf1Δ, reg1Δ, and reg1Δ snf1Δ mutant strains were grown at 25°C until exponential phase and treated with 4 mM dithiothreitol (DTT) for the indicated time. The mRNA levels were quantified by qRT-PCR analysis, and relative mRNA levels were calculated using ACT1 mRNA. The data show mean ± SEM (n = 3). *P < 0.05 and **P < 0.01 as determined by Student’s t-test. (TIF) [file pgen.1005491.s007.tif]

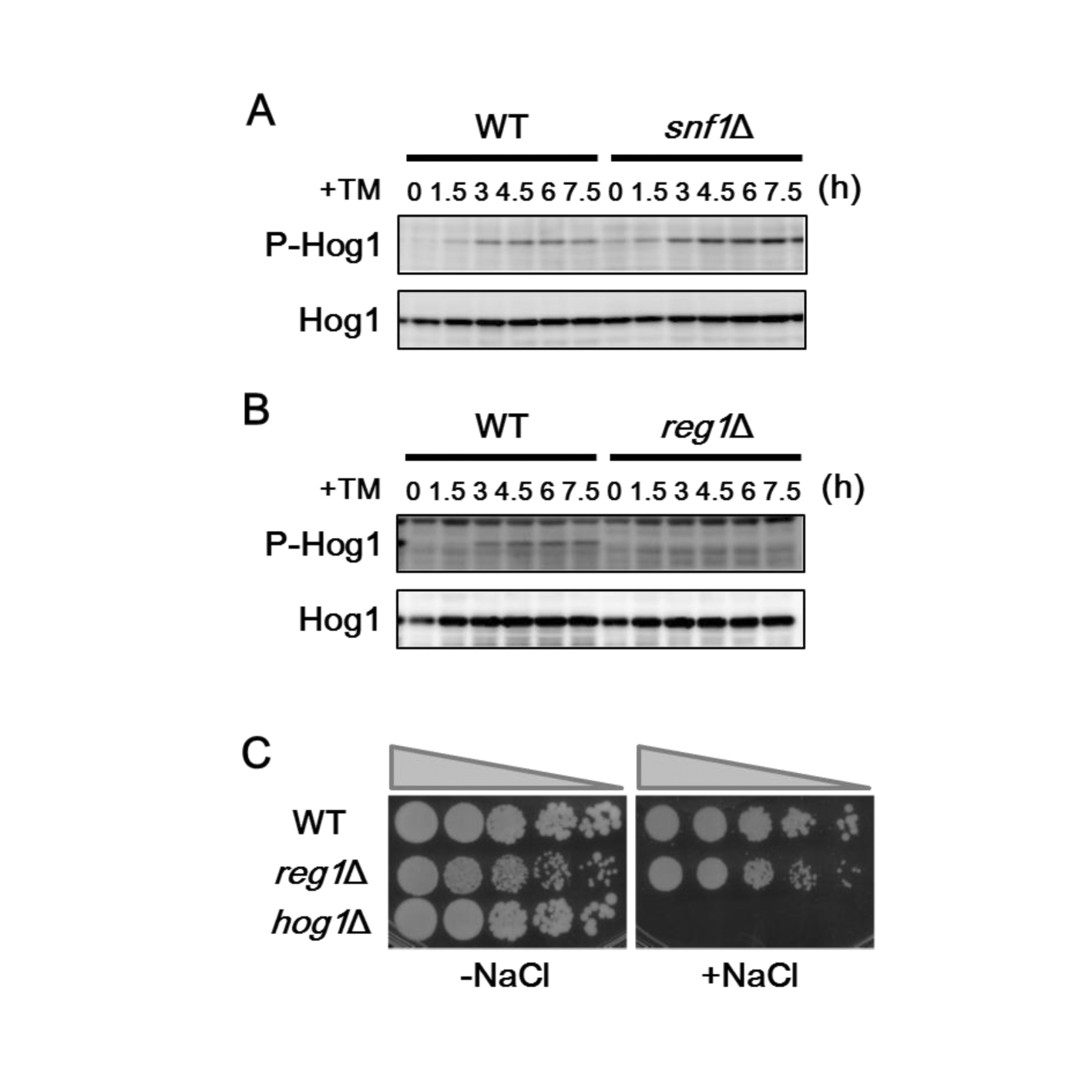

Supplement: S6 Fig — (A) Effects of the snf1Δ mutation on ER stress-induced Hog1 activation. Wild-type (WT) and snf1Δ mutant strains were grown at 25°C until exponential phase and treated with 2 μg/ml tunicamycin (TM) for the indicated time. Extracts prepared from each cell were immunoblotted with anti-phospho-p38 (P-Hog1) and anti-Hog1 antibodies. (B) Effects of the reg1Δ mutation on ER stress-induced Hog1 activation. Wild-type (WT) and reg1Δ mutant strains were analyzed as described in (A). (C) Osmotic stress sensitivity in the reg1Δ mutants. Wild-type (WT) and reg1Δ and hog1Δ mutant strains were spotted onto YPD medium lacking or containing 1 M sodium chloride (NaCl) and incubated at 25°C. (TIF) [file pgen.1005491.s008.tif]

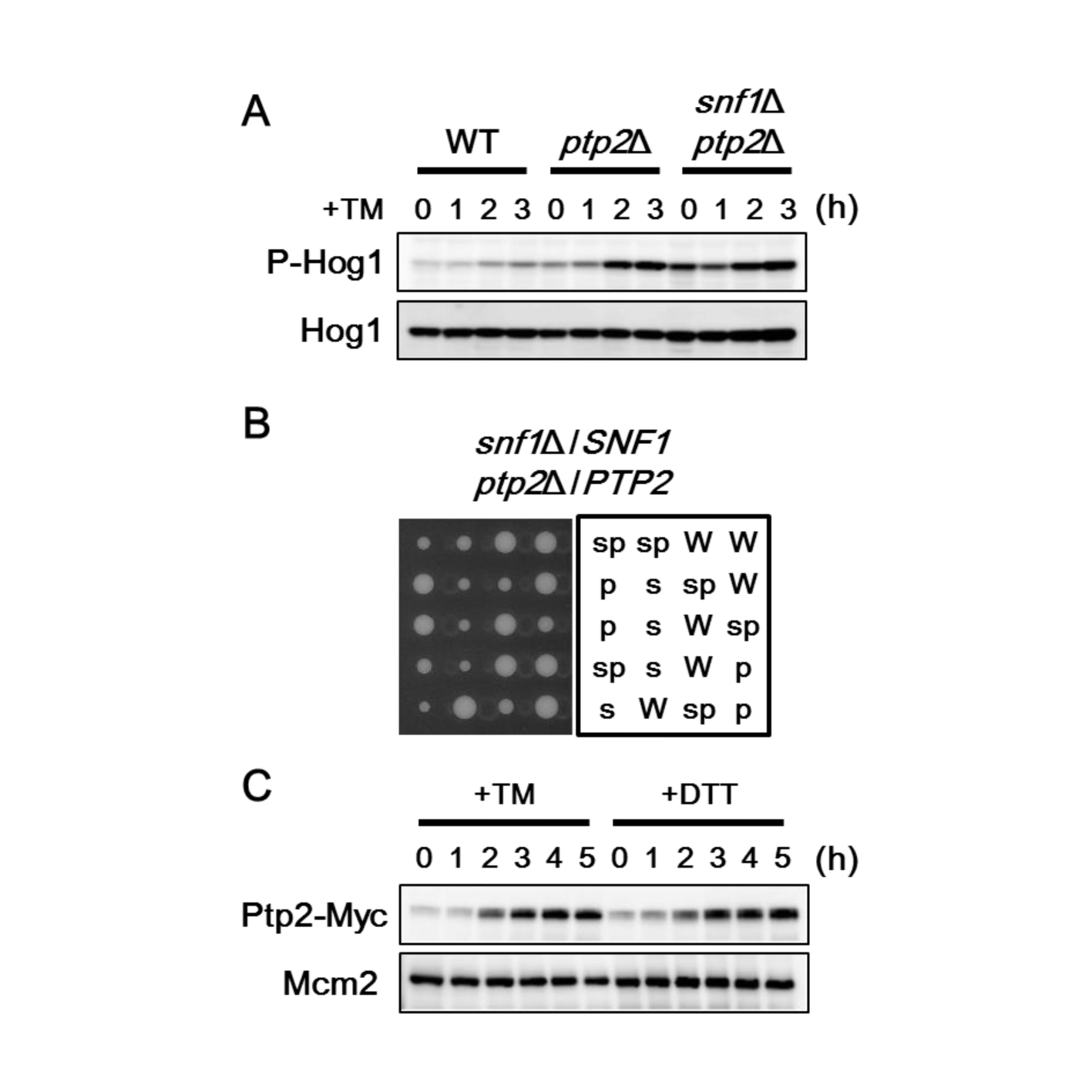

Supplement: S7 Fig — (A) Effects of the ptp2Δ snf1Δ mutations on ER stress-induced Hog1 activation. Wild-type (WT) and ptp2Δ, and ptp2Δ snf1Δ mutant strains were grown at 25°C until exponential phase and treated with 2 μg/ml tunicamycin (TM) for the indicated time. Extracts prepared from each cell were immunoblotted with anti-phospho-p38 (P-Hog1) and anti-Hog1 antibodies. (B) Genetic interaction between the snf1Δ and ptp2Δ mutations. Diploid snf1Δ/SNF1 ptp2Δ/PTP2 yeast cells were sporulated, dissected on YPD plates and the meiotic products were incubated at 25°C. Each genotype was shown in the right panel. Wild-type and snf1Δ and ptp2Δ mutant cells were labeled with W, s, and p, respectively. (C) The expression level of Ptp2 during ER stress response. Wild-type (WT) cells harboring harboring Myc-tagged PTP2 were grown at 25°C until exponential phase and treated with 2 μg/ml tunicamycin (TM) or 4 mM dithiothreitol (DTT) for the indicated time. Extracts prepared from each cell were immunoblotted with anti-Myc and anti-Mcm2 antibodies. (TIF) [file pgen.1005491.s009.tif]

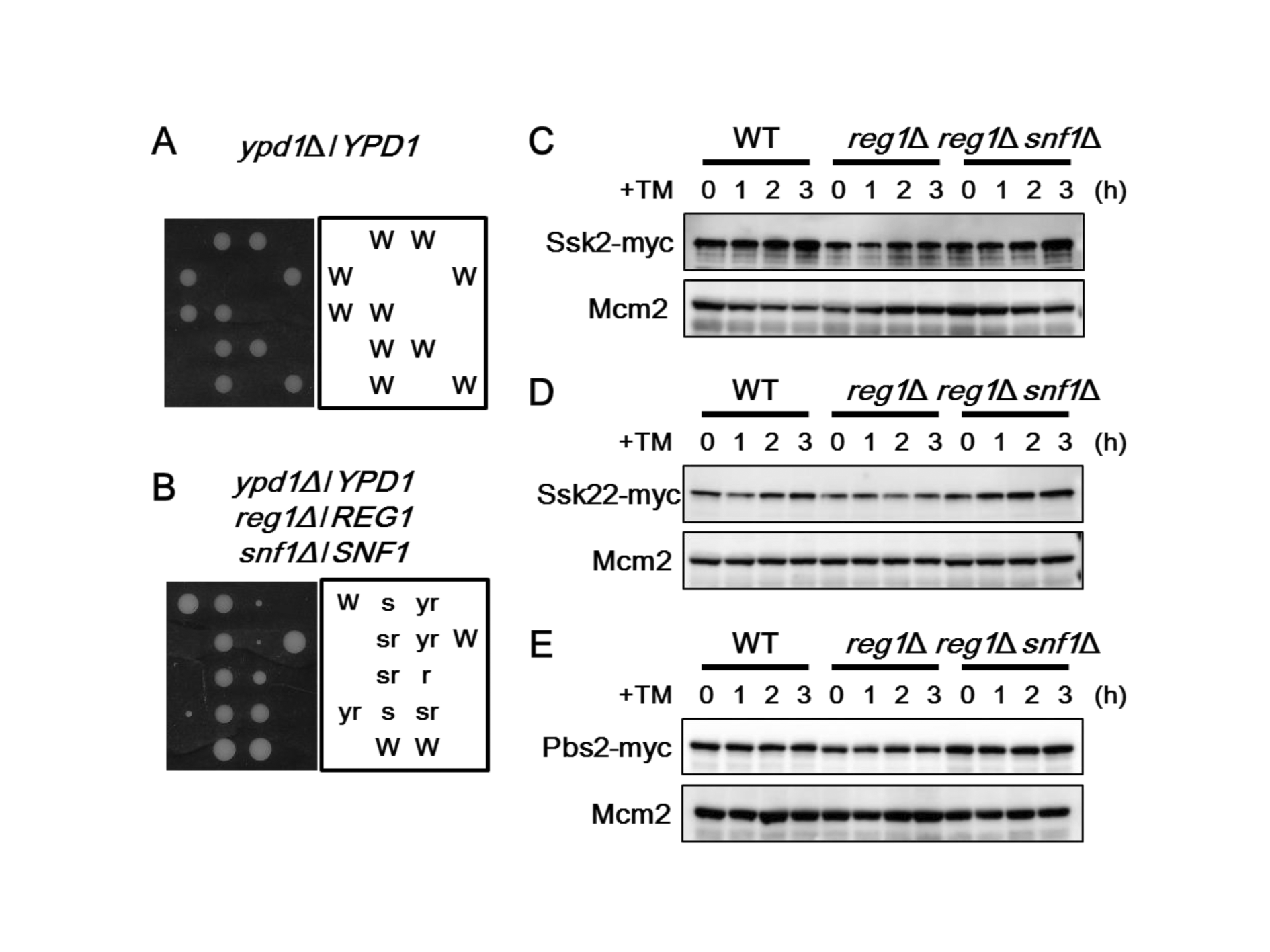

Supplement: S8 Fig — (A, B) Genetic interaction between the ypd1Δ, reg1Δ and snf1Δ mutations. Diploid ypd1Δ/YPD1 (A) and ypd1Δ/YPD1 reg1Δ/REG1 snf1Δ/SNF1 (B) yeast cells were sporulated, dissected on YPD plates and the meiotic products were incubated at 25°C. Each genotype was shown in the right panel. Wild-type and snf1Δ, reg1Δ, and ypd1Δ mutant cells were labeled with W, s, r, and y, respectively. (C-E) The expression levels of Ssk2, Ssk22 and Pbs2 during ER stress response. Wild-type (WT) and reg1Δ, and reg1Δ snf1Δmutant strains harboring Myc-tagged SSK2 (C), SSK22 (D), or PBS2 (E) were grown at 25°C until exponential phase and treated with 2 μg/ml tunicamycin (TM) for the indicated time. Extracts prepared from each cell were immunoblotted with anti-Myc and anti-Mcm2 antibodies. (TIF) [file pgen.1005491.s010.tif]

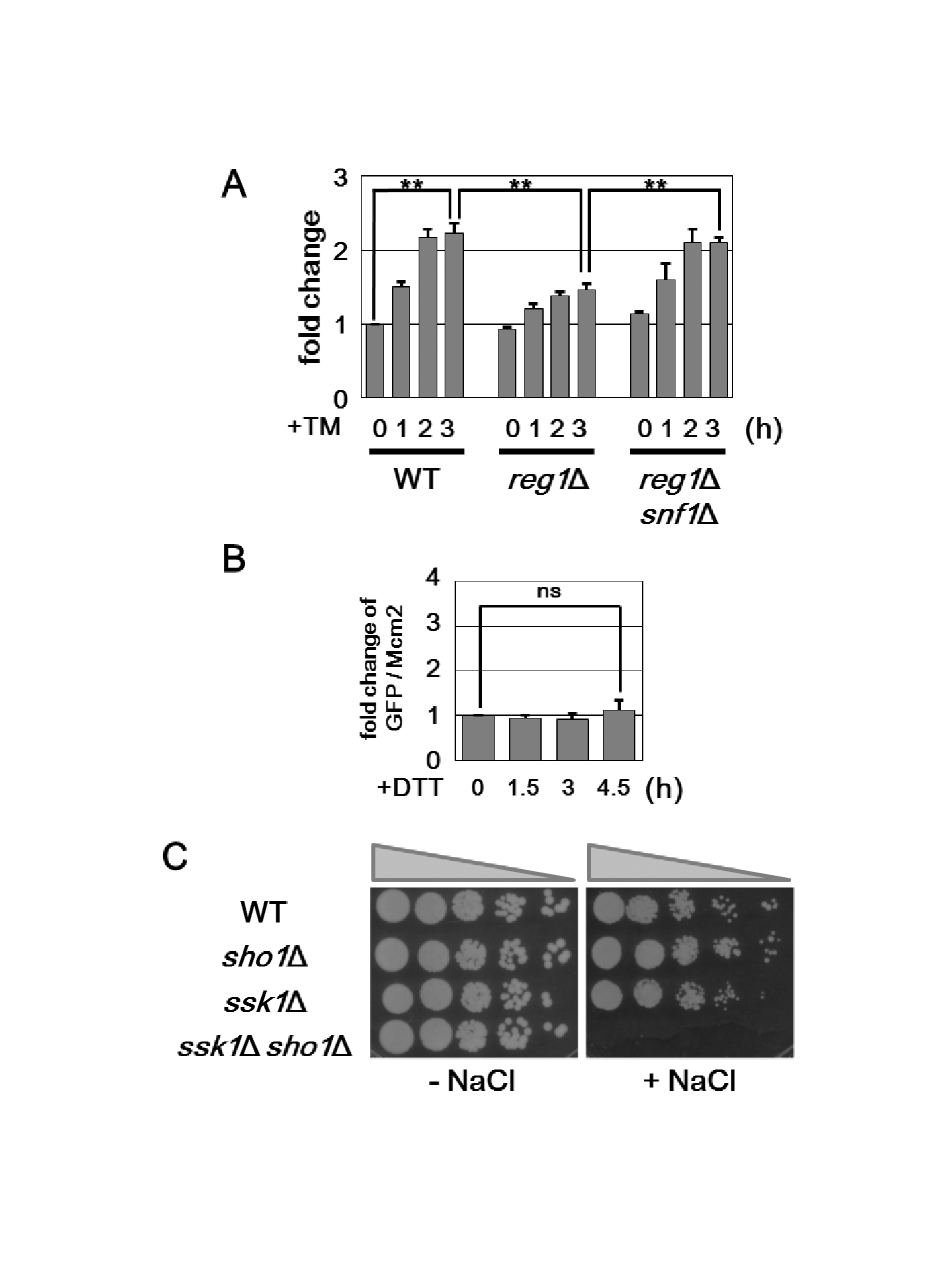

Supplement: S9 Fig — (A) Effects of the snf1Δ and reg1Δ mutations on ER stress-induced upregulation of SSK1 mRNA. Wild-type (WT) and reg1Δ and reg1Δ snf1Δ mutant strains were grown at 25°C until exponential phase and treated with 2 μg/ml tunicamycin (TM) for the indicated time. The mRNA levels were quantified by qRT-PCR analysis, and relative mRNA levels were calculated using ACT1 mRNA. The data show mean ± SEM (n = 4). **P < 0.01 as determined by Student’s t-test. (B) Effects of ER stress on the MCM2 promoter activity. Wild-type strain harboring a P MCM2 -GFP reporter plasmid were analyzed as described in Fig 6C. The intensities of GFP were measured and normalized to Mcm2 level. The values are plotted as the fold change from wild-type cells at the time of DTT addition. The data show mean ± SEM (n = 3). The statistical difference was determined by Student’s t-test. ns, not significant. (C) Osmotic stress sensitivity in the sho1Δ and ssk1Δ mutants. Wild-type (WT) and sho1Δ, ssk1Δ, and ssk1Δ sho1Δ mutant strains were spotted onto YPD medium lacking or containing 1 M sodium chloride (NaCl) and incubated at 25°C. (TIF) [file pgen.1005491.s011.tif]
